# Adaptive immunity and neutralizing antibodies against SARS-CoV-2 variants of concern following vaccination in patients with cancer: The CAPTURE study

**DOI:** 10.1038/s43018-021-00274-w

**Published:** 2021-10-27

**Authors:** Annika Fendler, Scott T.C. Shepherd, Lewis Au, Katalin A. Wilkinson, Mary Wu, Fiona Byrne, Maddalena Cerrone, Andreas M. Schmitt, Nalinie Joharatnam-Hogan, Benjamin Shum, Zayd Tippu, Karolina Rzeniewicz, Laura Amanda Boos, Ruth Harvey, Eleanor Carlyle, Kim Edmonds, Lyra Del Rosario, Sarah Sarker, Karla Lingard, Mary Mangwende, Lucy Holt, Hamid Ahmod, Justine Korteweg, Tara Foley, Jessica Bazin, William Gordon, Taja Barber, Andrea Emslie-Henry, Wenyi Xie, Camille L. Gerard, Daqi Deng, Emma C. Wall, Ana Agua-Doce, Sina Namjou, Simon Caidan, Mike Gavrielides, James I MacRae, Gavin Kelly, Kema Peat, Denise Kelly, Aida Murra, Kayleigh Kelly, Molly O’Flaherty, Lauren Dowdie, Natalie Ash, Firza Gronthoud, Robyn L. Shea, Gail Gardner, Darren Murray, Fiona Kinnaird, Wanyuan Cui, Javier Pascual, Simon Rodney, Justin Mencel, Olivia Curtis, Clemency Stephenson, Anna Robinson, Bhavna Oza, Sheima Farag, Isla Leslie, Aljosja Rogiers, Sunil Iyengar, Mark Ethell, Christina Messiou, David Cunningham, Ian Chau, Naureen Starling, Nicholas Turner, Liam Welsh, Nicholas van As, Robin L. Jones, Joanne Droney, Susana Banerjee, Kate C. Tatham, Mary O’Brien, Kevin Harrington, Shreerang Bhide, Alicia Okines, Alison Reid, Kate Young, Andrew J.S. Furness, Lisa Pickering, Charles Swanton, on behalf of the Crick COVID19 consortium, Sonia Gandhi, Steve Gamblin, David LV Bauer, George Kassiotis, Sacheen Kumar, Nadia Yousaf, Shaman Jhanji, Emma Nicholson, Michael Howell, Susanna Walker, Robert J. Wilkinson, James Larkin, Samra Turajlic

**Affiliations:** 1 Cancer Dynamics Laboratory, The Francis Crick Institute, London, NW1 1AT, UK; 2 Skin and Renal Units, The Royal Marsden NHS Foundation Trust, London, SW3 6JJ, UK; 3 Tuberculosis Laboratory, The Francis Crick Institute, London, NW1 1AT, UK; 4 Wellcome Center for Infectious Disease Research in Africa, University of Cape Town, Observatory, Cape Town, Republic of South Africa; 5 High Throughput Screening Laboratory, The Francis Crick Institute, London, NW1 1AT, UK; 6 Department of Infectious Disease, Imperial College London, London, UK; 7 Worldwide Influenza Centre, The Francis Crick Institute, London, NW1 1AT, UK; 8 Haemato-oncology Unit, The Royal Marsden NHS Foundation Trust, London, SW3 6JJ, UK; 9 University College London Hospitals NHS Foundation Trust Biomedical Research Centre, London, NW1 1AT, UK; 10 Structural Biology of Disease Processes Laboratory, The Francis Crick Institute, London, NW1 1AT, UK; Experimental Histopathology Laboratory, The Francis Crick Institute, London, NW1 1AT, UK; 11 Flow Cytometry Scientific Technology Platform, The Francis Crick Institute, London, NW1 1AT, UK; 12 Safety, Health & Sustainability, The Francis Crick Institute, London, NW1 1AT, UK; 13 Scientific Computing Scientific Technology Platform, The Francis Crick Institute, London, NW1 1AT, UK; 14 Metabolomics Scientific Technology Platform, The Francis Crick Institute, London, NW1 1AT, UK; 15 Department of Bioinformatics and Biostatistics, The Francis Crick Institute, London, UK; 16 Department of Pathology, The Royal Marsden NHS Foundation Trust, London, NW1 1AT, UK; 17 Translational Cancer Biochemistry Laboratory, The Institute of Cancer Research, London, SW7 3RP, UK; 18 Clinical Trials Unit, The Royal Marsden NHS Foundation Trust, London, SM2 5PT, UK; 19 Lung Unit, The Royal Marsden NHS Foundation Trust, London, SW3 6JJ, UK; 20 Breast Unit, The Royal Marsden NHS Foundation Trust, London, SW3 6JJ, UK; 21 Gastrointestinal Unit, The Royal Marsden NHS Foundation Trust, London and Surrey SM2 5PT; 22 Department of Radiology, The Royal Marsden NHS Foundation Trust, London, SW3 6JJ, UK; 23 Neuro-oncology Unit, The Royal Marsden NHS Foundation Trust, London, SW3 6JJ, UK; 24 Clinical Oncology Unit, The Royal Marsden NHS Foundation Trust, London, SW3 6JJ, UK; 25 Sarcoma Unit, The Royal Marsden NHS Foundation Trust and Institute of Cancer Research, London, SW3 6JJ, UK; 26 Palliative Medicine, The Royal Marsden NHS Foundation Trust, London, SW3 6JJ, UK; 27 Gynaecology Unit, The Royal Marsden NHS Foundation Trust, London, SW3 6JJ, UK; 28 Anaesthetics, Perioperative Medicine and Pain Department, The Royal Marsden NHS Foundation Trust, London, SW3 6JJ, UK; 29 Head and Neck, The Royal Marsden NHS Foundation Trust, London, SW3 6JJ, UK; 30 Targeted Therapy Team, The Institute of Cancer Research, London, SW7 3RP, UK; 31 Acute Oncology Service, The Royal Marsden NHS Foundation Trust, London, SW3 6JJ, UK; 32 Uro-oncology unit, The Royal Marsden NHS Foundation Trust, Surrey, SM2 5PT; 33 Cancer Evolution and Genome Instability Laboratory, The Francis Crick Institute, London, NW1 1AT, UK; 34 University College London Cancer Institute, London WC1E 6DD, UK; 35 Neurodegeneration Biology Laboratory, The Francis Crick Institute, London, NW1 1AT, UK; 36 UCL Queen Square Institute of Neurology, Queen Square, London WC1N 3BG; 37 RNA Virus Replication Laboratory, The Francis Crick Institute, London, NW1 1AT, UK; 38 Retroviral Immunology Laboratory, The Francis Crick Institute, London, NW1 1AT, UK

**Keywords:** SARS-CoV-2, COVID-19, Cancer, Adaptive Immunity, Antibody Response, Neutralising Antibodies, T-cell Response, Prospective Study, Vaccine

## Abstract

CAPTURE (NCT03226886) is a prospective cohort study of COVID-19 immunity in patients with cancer. Here we evaluated 585 patients following administration of two doses of BNT162b2 or AZD1222 vaccines, administered 12 weeks apart. Seroconversion rates after two doses were 85% and 59% in patients with solid and hematological malignancies, respectively. A lower proportion of patients had detectable neutralizing antibody titers (NAbT) against SARS-CoV-2 variants of concern (VOCs) vs wildtype (WT). Patients with hematological malignancies were more likely to have undetectable NAbT and had lower median NAbT vs solid cancers against both WT and VOCs. In comparison with individuals without cancer, patients with haematological, but not solid, malignancies had reduced NAb responses. Seroconversion showed poor concordance with NAbT against VOCs. Prior SARS-CoV-2 infection boosted NAb response including against VOCs, and anti-CD20 treatment was associated with undetectable NAbT. Vaccine-induced T-cell responses were detected in 80% of patients, and were comparable between vaccines or cancer types. Our results have implications for the management of cancer patients during the ongoing COVID-19 pandemic.

## Introduction

Ongoing evolution of the severe acute respiratory syndrome coronavirus 2 (SARS-CoV-2) has led to the emergence of variants of concern (VOC) that have potentially enhanced transmission, pathogenicity, and immune escape^1^. Additionally, mutations affecting spike epitopes could reduce protection induced by vaccines developed based on wild-type (WT) spike. The highly infectious Delta VOC (B.1.167.2), first identified in India in early 2021, is currently the predominant variant worldwide. Despite its surging prevalence, it is suggested that vaccination programmes have broken the link between infection and hospitalisation and death^2^, with many countries lifting COVID-19 restrictions. In the United Kingdom (UK), however, those classified as clinically extremely vulnerable are still advised to take additional precautions of their own accord ^3^, without clear messaging regarding immune response to vaccines or vaccine efficacy around individual conditions within this heterogeneous clinical group. Furthermore, vulnerable patients were shown to be disproportionately affected by vaccine breakthrough infections^4^. In one study of 152 double-vaccinated patients hospitalized due to COVID-19, 40% were immunosuppressed (19% chronic corticosteroid treatment, 18% chemotherapy/antimetabolite treatment, 11% solid organ transplant, 7% anti-CD20 treatment), and overall cohort mortality was 22%^5^. Recently, preliminary results on BNT162b2 and AZD1222 vaccine effectiveness in extremely clinically vulnerable patients in England showed strong S-reactive antibody response and vaccine effectiveness against symptomatic COVID-19 in all vulnerable groups except the immunocompromised, particularly after a single dose^6^.

Patients with cancer represent an important vulnerable group (estimated 19.3 million new cancer diagnosis per year globally^7^), with an increased likelihood of poor clinical outcomes from COVID-19^8–11^. As such, cancer patients have been prioritised in COVID-19 vaccination programmes globally^12, 13^, however, as they were virtually excluded from the pivotal vaccine studies, data on efficacy or immune response to COVID-19 vaccines in this population are lacking. Given that cancer or its treatment may impact immunity, characterisation of immune response to COVID-19 vaccines in cancer patients represents a priority. Available studies demonstrated generally high seroconversion rates after two vaccine doses in patients with solid cancers (≥90%, measured as IgG),^14–17^ with less pronounced responses in those with haematological malignancies (compounded by treatments including anti-CD20 therapy)^14, 18–23^. However, data on the functionally relevant SARS-CoV-2 neutralising antibody responses, particularly to VOC, are scarce. Vaccine-induced T-cell responses have been reported in cancer patients^15, 24^, but, again, activity against VOCs is unknown. Furthermore, although humoral and cellular responses to SARS-CoV-2 often correlate^25^, this has not been assessed regarding COVID-19 vaccines, nor investigated in cancer patients specifically. Finally, the impact of prior infection on subsequent vaccine-induced immunity in cancer patients remains unclear. In the context of emerging VOC, such data are urgently needed to calibrate risk-mitigation measures and tailor vaccine regimes for cancer patients.

CAPTURE (COVID-19 antiviral response in a pan-tumour immune monitoring study) is a prospective, longitudinal cohort study evaluating the impact of cancer and anti-cancer treatment on the immune response to SARS-CoV-2 infection and COVID-19 vaccinations^26^. Data from the infection cohort (companion paper Fendler et al.) show that the majority of solid cancer patients develop durable humoral responses (of at least 11 months), and have detectable T-cell responses to SARS-CoV- 2 infection, but patients with haematological malignancies often display a discordance between the humoral and cellular arms (owing to disease-related lineage defects and anti-CD20 treatment); additionally, neutralising activity against the Alpha, Beta and Delta VOCs is reduced following infection with the WT SARS-CoV-2 strain. Here, we investigate whether humoral and cellular immunity is efficiently induced following COVID-19 vaccination in the vaccine cohort of the CAPTURE study, especially regarding VOCs. Of note, the study is conducted in the UK, where vaccination schedule initially followed an off-label 12-week between doses interval. This approach was implemented by the UK government during the second wave of the pandemic to maximise the number of people vaccinated with at least one dose.

## Results

### Cohort characteristics and COVID-19 vaccination

Between May 2020 and June 2021 (database lock), we recruited 626 cancer patients who received at least one COVID-19 vaccine dose, of whom 41 (7%) had no follow-up samples and were excluded from the analysis (Extended Data Figure 1a, Table 1). Of the 585 evaluable patients, 93% received two vaccine doses; 74% (430/585) received the AZD1222 vaccine (Oxford/AstraZeneca, [AZ]) and 26% (153/585) the BNT162b2 (Pfizer Biontech, [PZ]). Overall, 93% (546/585) received the second dose, at a median of 77 days (IQR 72-78) in accordance with the 12-week interval in-between vaccine doses guidance^13^. Five percent of patients (29/585) did not receive a second vaccine dose due to either cancer-related death (3%, 16/585), clinical advice (1%, 7/585) or patient preference (1%, 6/585), and 2% of patients (10/585) either withdrew study consent or were lost to follow-up (Table 1, Extended Data Figure 1a). There were no baseline differences between patients who were included or excluded from the final analysis, nor between patients receiving one or two vaccine doses (Supplementary Table 1). Restrictions on hospital attendance during the pandemic resulted in a small number of missed follow-up samples (Extended Data Figure 1b, Supplementary Table 2).

The median age of patients was 60 years (IQR: 52-68), and 60% (323/585) were male. Overall, 69% of patients (404/585) were SARS-CoV-2 infection-naive and 31% (181/585) had prior SARS-CoV-2 infection confirmed either by SARS-CoV-2 RT-PCR (median time from positive test to baseline of 77 days [IQR: 40-287]), or presence of S1-reactive antibodies at baseline. In total, 76% of patients (447/585) had a current diagnosis of solid and 24% (138/585) of haematological malignancy. The majority of patients with solid cancers had metastatic disease (68% [306/447]) (Table 1). Patients receiving PZ were more likely to be older (median 63 vs 59 years AZ, p<0.001) and have a haematological malignancy (35% vs 23% AZ, p=0.02) reflecting earlier licensing of PZ and prioritisation of these groups as extremely clinically vulnerable. Patients with haematological malignancies were more likely to be male (60% vs 55% solid cancers, p=0.01) and patients receiving PZ were more likely to have had prior SARS-Cov2 infection (Supplementary Table 3).

Overall, 21% of patients (123/585) received treatment with chemotherapy, 34% (200/585) with targeted therapy, and 3% (20/585) with endocrine therapy within 28 days prior to vaccination. Thirty-one percent of patients (185/585) received immune-checkpoint inhibitors (CPI), with 19% of patients (109/585) receiving CPI treatment within 183 days of vaccination; 22% (24/109) had active immune related adverse events (irAEs) secondary to CPI, although only 4% (4/109) received immunosuppression with corticosteroids (>10mg prednisolone equivalent for ≥7 days) within 48 hours of vaccination.

In total, 4% (26/585) received anti-CD20 therapy within 12 months of vaccination and 10% (58/585) previously received haematological stem cell transplant (43% allogeneic [25/58]; 57% autologous [33/58]), of which 16% (9/58) were within six months of vaccination; 31% (18/58) had active graft-versus-host-disease (GvHD) requiring immunosuppression at the time of vaccination. Five percent (32/585) had radiotherapy or surgery within 28 days of vaccination.

At the time of vaccination, 7% of patients (39/585) were receiving immunosuppressive therapy with corticosteroids (5% [29/585]; >10mg prednisolone equivalent for ≥7 days) and/or other immunosuppressive therapy (2% [14/585]) including tacrolimus, methotrexate, cyclosporine and mycophenolate mofetil.

### Seroconversion rates following COVID-19 vaccines

**Figure 1:**
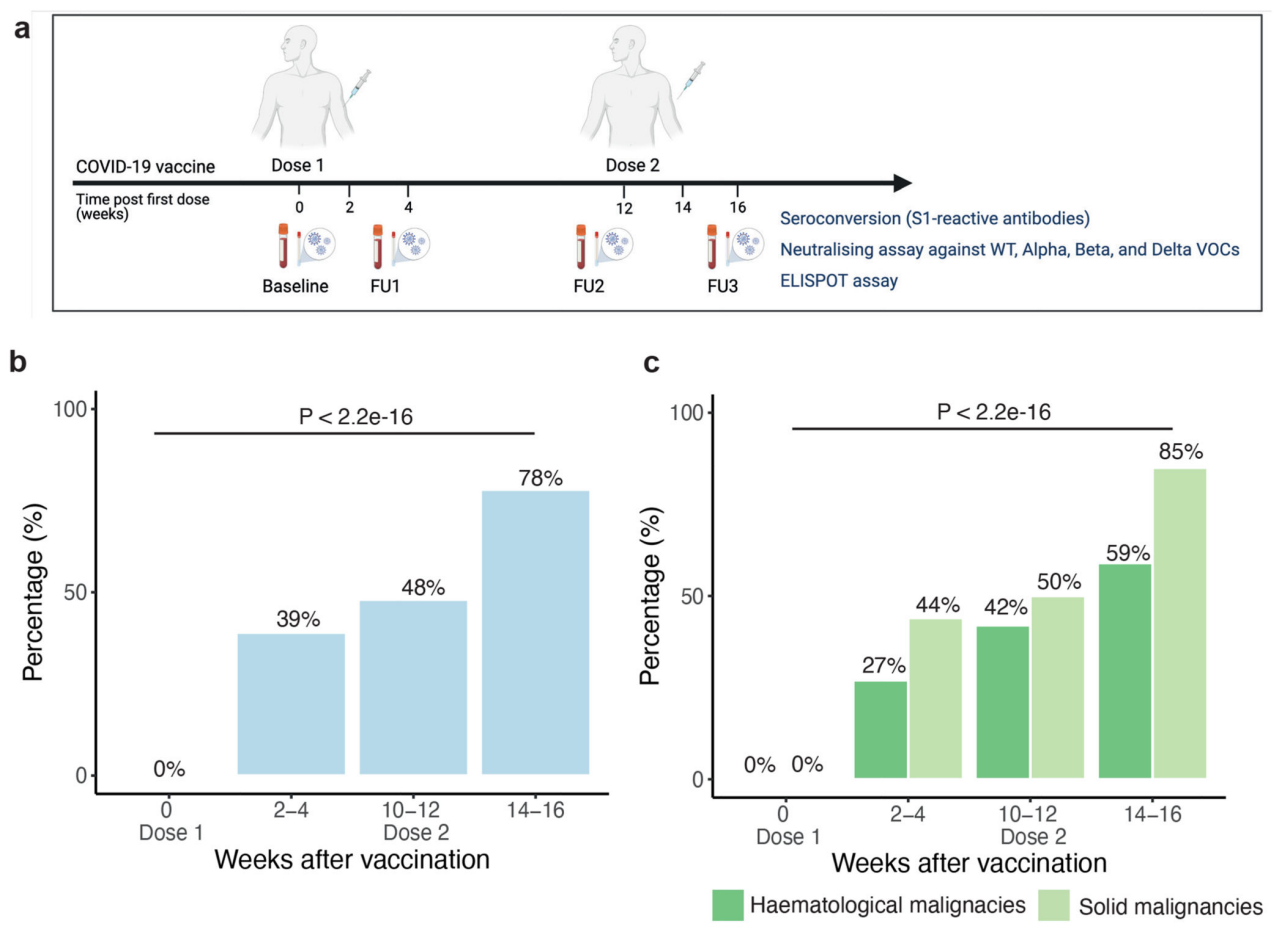
Seroconversion in cancer patients after COVID-19 vaccination **a)** Sampling and analysis schema within the CAPTURE study. Baseline samples were collected immediately before the first dose. Follow-up samples were collected: 2-4 weeks post-first dose (FU1), on the day and immediately before the second dose (FU2; ie, the additional post-first dose timepoint implemented due to delayed 12 week dosing interval), and 2-4 weeks post-second dose (FU3). S1-reactive antibody (i.e., seroconversion) and neutralising antibody assays were performed in all available follow-up samples from 585 patients. **b)** Proportion of infection naive patients (n= 328/323/256/312 patients at BL/FU1/FU2/FU3) with S1-reactive antibodies at each timepoint. Differences were analysed using Chi-Square test. p-values < 0.05 were considered significant. **c)** proportion of infection patients with S1-reactive Ab grouped by solid (n= 270/234/192/234 patients at BL/FU1/FU2/FU3) and haematological malignancies (n=58/89/64/78 patients at BL/FU1/FU2/FU3). Differences were analysed by the Chi-Square test. p-values < 0.05 were considered significant. Ab, antibodies; BL, baseline; FU1, 21-56 days post first-vaccine; FU2, 14-28 days prior to second-vaccine; FU3, 14-28days post second-vaccine

Seroconversion (i.e. presence of S1-reactive antibodies) was assessed in infection-naive patients (defined as no history of COVID-19; negative SARS-CoV-2 RT-PCR, and negative S1-reactive antibodies prior to vaccination) at baseline, 2-4 weeks post-first, and 2-4 weeks post-second vaccine dose (Figure 1a). Due to the uncertainty of the effect of the extended 12-week dosing interval, we incorporated an additional sampling timepoint just prior to the second vaccine dose (Figure 1a). Post-first dose, the seroconversion rate was 39% (Figure 1b), with lower rates in patients with haematological (27%) vs solid malignancies (44%) (Figure 1c). Post-second dose, this increased to 78%, again with lower rates for haematological (59%) vs solid malignancies (85%) (Figure 1b,c). Seroconversion rates were maintained during the 12-week dosing interval, with a nominal increase in the number of seroconverted patients just prior to the second dose relative to the earlier timepoint at 2-4 weeks (especially in those with haematological malignancies) (Figure 1b,c).

### NAb responses following COVID-19 vaccines

Functional humoral responses after vaccination were assessed in all patients using a high-throughput live-virus neutralisation assay (Methods) against WT SARS-CoV-2, and Alpha (B.1.1.7), Beta (B.1.351), and Delta (B.1.617.2) VOCs, and expressed as titres (representing the reciprocal of serum required to inhibit 50% of viral replication [IC50]). The distribution of neutralising antibody (NAb) titres (NAbT) was categorised as undetectable (<40), moderate (40-256), and high (>256), as per previously published reports using the same neutralisation assay ^27–29^.

**Figure 2:**
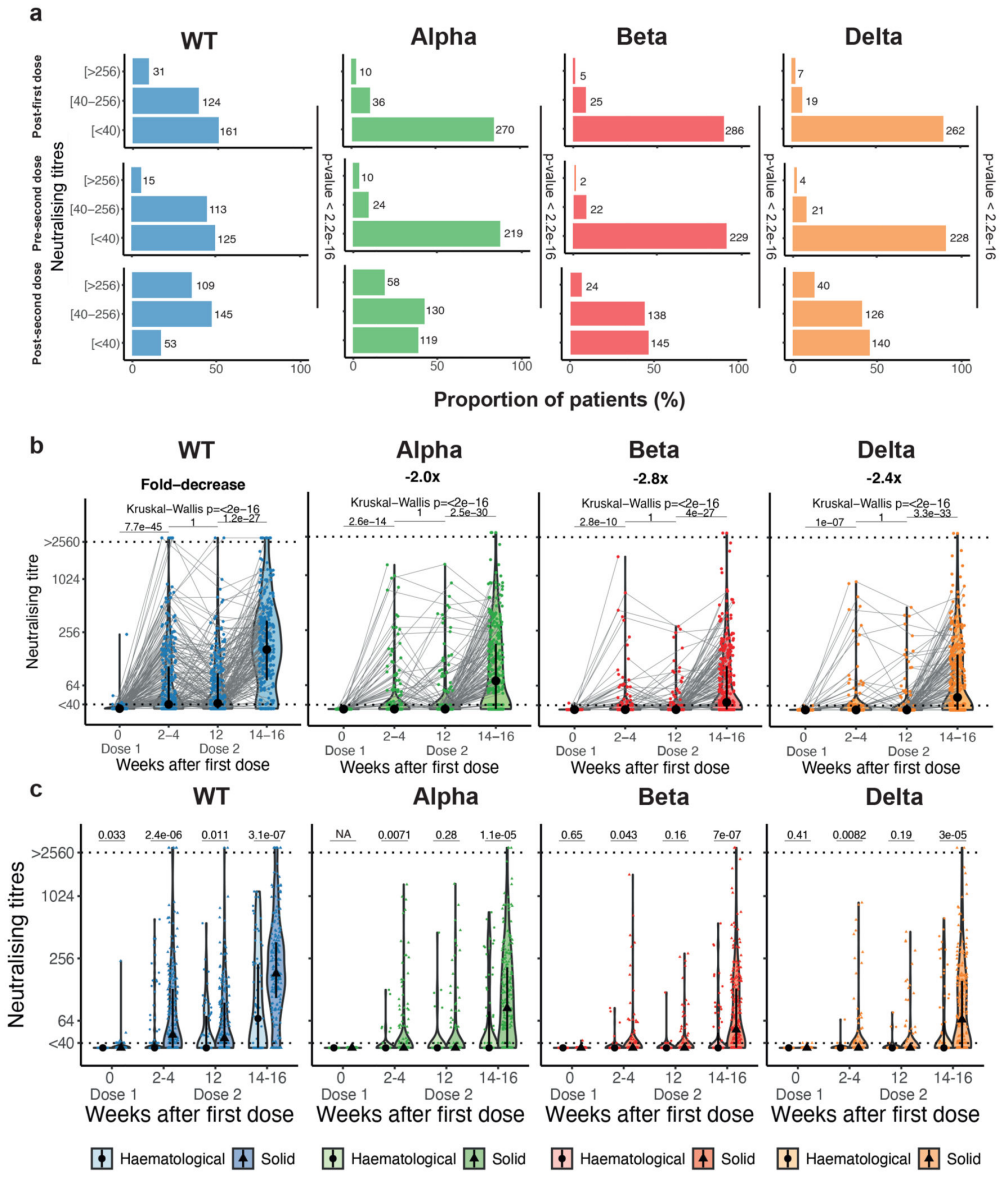
Neutralising antibodies against WT SARS-CoV-2 and VOCs **a)** NAbT in infection-naive patients were categorised as undetectable/low (<40), medium (40-256), or high (>256) are shown for WT SARS-CoV-2 and the three VOCs. Differences were analysed using Chi-Square test. p-values < 0.05 were considered significant. Numbers in the panel indicate sample numbers. **b)** NAbT in infection-naive patients against WT SARS-CoV-2, Alpha, Beta, and Delta VOCs. Median fold-decrease in NAbT is shown for each VOC in comparison to WT SARS-CoV-2 (n= 318/316/253/307 patients at BL/FU1/FU2/FU3). Dotted line at <40 denotes the lower limit of detection, dotted line at >2560 denotes the upper limit of detection. Violin plots denote density of data points. PointRange denotes the median and the 25 and 75 percentiles. Dots represent individual samples. Samples from individual patients are connected. Significance was tested by Kruskal Wallis test, p < 0.05 was considered significant, post-hoc test: two-sided Mann Whitney-U test with Bonferroni correction was used for pairwise comparisons. Only comparisons with the prior timepoint are denoted in the graph. **c)** Comparison of NAbT in infection-naive patients with solid (n= 262/232/189/232 patients at BL/FU1/FU2/FU3) vs haematological malignancies patients (n= 56/84/64/75 patients at BL/FU1/FU2/FU3). Dotted line at <40 denotes the lower limit of detection, dotted line at >2560 denotes the upper limit of detection. Violin plots denote density of data points. PointRange denotes the median and the 25 and 75 percentiles. Dots represent individual samples. Significance was tested by two-sided Wilcoxon-Mann-Whitney U test, p < 0.05 was considered significant. NAbT, neutralising antibody titre. NA, not tested. BL, baseline; FU1, 21-56 days post firstvaccine; FU2, 14-28 days prior to second-vaccine; FU3, 14-28days post second-vaccine.

Post-first dose, 49% of infection-naive patients had detectable NAb to WT SARS-CoV-2, with significantly lower proportion having detectable NAb to VOCs (Alpha 15%, Beta 9%, Delta 9%) (Figure 2a); the median NAbT were below the limit of detection for all strains (Figure 2b). Post-second dose, the proportion of patients with detectable NAbT against all strains increased, but less so against VOCs vs WT (WT 83%, Alpha 61%, Beta 53%, Delta 54%; Chi^2^-test, p-value < 2.2x10^-16^); the median NAbT also increased for all strains, again to a lesser extent for VOCs vs WT (Figure 2a,b).

**Figure 3:**
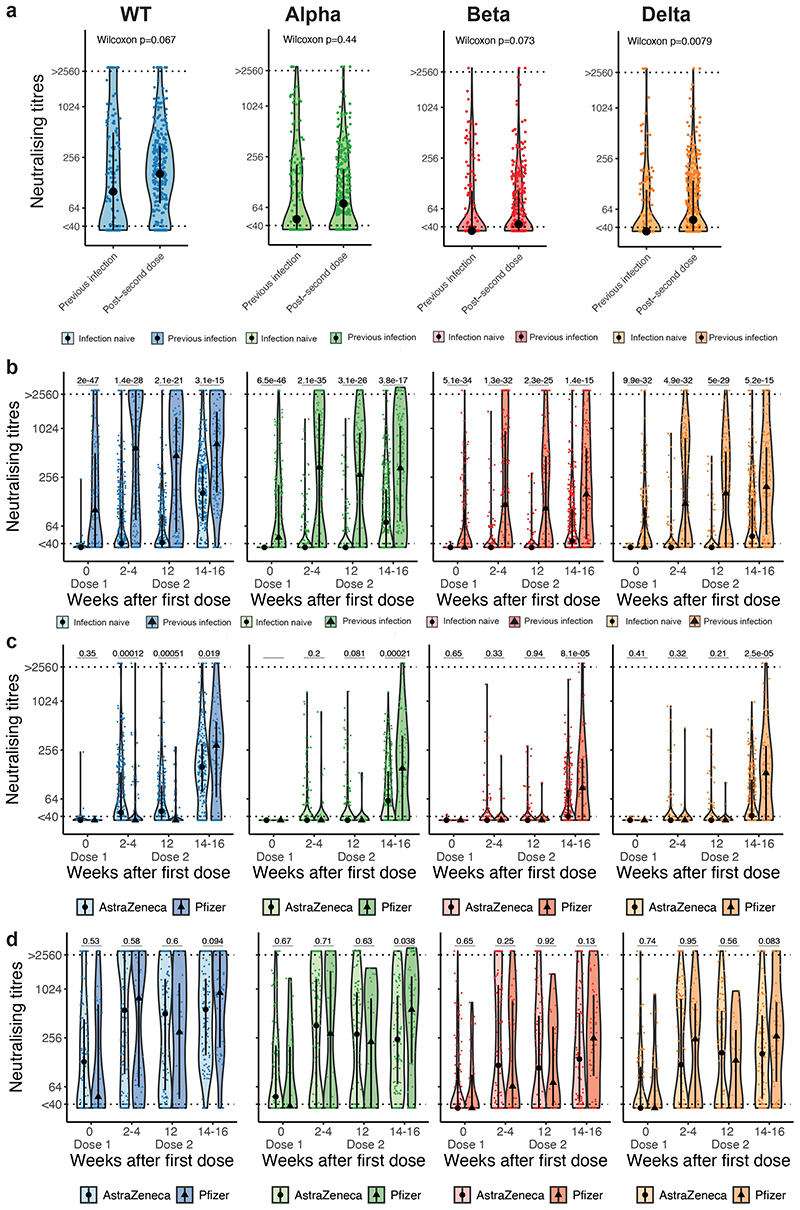
Neutralising response against WT SARS-CoV-2 and VOCs by prior SARS-CoV-2 infection status and type of COVID-19 vaccine **a)** Comparison of NAbT against WT SARS-CoV-2, Alpha, Beta, and Delta in patients with previous infection before vaccination vs infection naive patients post-second dose (n= 133/306 patients at BL/FU3). Significance was tested by two-sided Wilcoxon-Mann-Whitney U test, p < 0.05 was considered significant. **b)** Comparison of NAbT against WT SARS-CoV-2, Alpha, Beta, and Delta in infection naive (n= 318/316/253/307 patients at BL/FU1/FU2/FU3) vs patients previously infected with SARS-CoV-2 (n= 133/163/115/144 patients at BL/FU1/FU2/FU3). **c)** Comparison of NAbT against WT SARS-CoV-2, Alpha, Beta, and Delta in infection-naive patients receiving AZ (n= 262/246/212/229 patients at BL/FU1/FU2/FU3) vs PZ (n= 56/70/41/77 patients at BL/FU1/FU2/FU3, 1 patient with unknown vaccine type not included), and **d)** in patients with previous SARS-CoV-2 infection receiving AZ (n= 99/117/92/91 patients at BL/FU1/FU2/FU3) vs PZ (n=34/46/23/53) patients at BL/FU1/FU2/FU3). Dotted line at <40 denotes the lower limit of detection, dotted line at >2560 denotes the upper limit of detection. Violin plots denote density of data points. PointRange denotes the median and the 25 and 75 percentiles. Dots represent individual samples. Significance in b-d was tested by two sided Wilcoxon-Mann-Whitney U test, p < 0.05 was considered significant. AZ, AstraZeneca; NAbT, neutralising antibody titres; PZ, Pfizer; VOC, variant of concern. NA, not tested. BL, baseline; FU1, 21-56 days post first-vaccine; FU2, 14-28 days prior to second-vaccine; FU3, 1428days post second-vaccine.

We observed that NAbT against Delta, but not against WT, Alpha, or Beta, were significantly higher in infection-naive patients who received two vaccine doses than in vaccine-naive patients who recovered from SARS-CoV-2 (WT or Alpha) infection (Figure 3a). Among those with prior SARS-CoV-2 infection, the proportion of patients with NAb against WT SARS-CoV-2 increased from 62% at baseline to 85% post-first, and 95% post-second vaccine dose (the corresponding proportions for Alpha were 52%, 65%, and 88%; Beta 39%, 61%, and 80%; Delta 41%, 59%, and 80%). Post-first and post-second dose, patients with prior SARS-CoV-2 infection had significantly higher median NAbT vs infection-naive patients (WT: 15-fold post-first, and 4-fold post-second dose; Alpha: 10-fold, and 5-fold; Beta: 3-fold and 4-fold; Delta: 3-fold and 4-fold) (Figure 3b).

We next analysed NAb response by vaccine type. In SARS-CoV-2 infection-naive patients, there were no significant differences in the proportion of patients with detectable NAb by vaccine type post-first dose (AZ: 54% WT, 16% Alpha, 10% Beta, 10% Delta; PZ: 30% WT, 9% Alpha, 7% Beta, 5% Delta), with higher median NAbT observed for AZ vs PZ against WT SARS-CoV-2 but not VOCs (Figure 3c). Post-second dose, there were significant differences in the proportion of patients with detectable NAb by vaccine type (AZ: 85% WT, 59% Alpha, 49% Beta, 50% Delta; PZ: 78% WT, 68% Alpha, 64% Beta, 68% Delta), with significantly lower median NAbT observed for AZ vs PZ against all variants (Figure 3c). In patients with previous SARS-CoV-2 infection, there were no differences in the proportion with detectable NAb post-second dose (AZ: 96% WT, 88% Alpha, 78% Beta, 79% Delta; PZ: 92% WT, 86% Alpha, 83% Beta, 81% Delta), or median NAbT post-first dose against all variants, nor post-second dose against WT, Alpha and Beta VOCs. However, post-second dose NAbT against Delta were significantly higher with PZ vs AZ (Figure 3d).

### NAb against VOC and S1-reactive antibodies show discordance

To understand the ability of the S1-reactive antibody assay (detecting IgG antibodies against WT SARS-CoV-2) to predict functional humoral immunity against currently circulating VOCs, we analysed per patient agreement of seroconversion and detectable NAbs. We observed good concordance between the presence of S1-reactive antibodies and NAb against the WT strain. However, there was discordance in the case of VOCs, e.g. 55% of patients without detectable NAb against Delta had detectable anti-S1 IgG Ab following two vaccine doses (Supplementary Table 4).

### Impact of prior infection and cancer subtypes on NAb response

Among infection-naive patients with solid cancers (n=308), post-first dose, 58% had detectable NAb against WT SARS-CoV-2, 17% against Alpha, 11% against Beta, and 12% against Delta. Post-second dose, this increased to 92% against WT, 70% against Alpha, 61% against Beta and 62% against Delta. However, in infection-naive patients with haematological malignancies (n=96) the proportions were lower both post-first (25% WT, 7% Alpha, 5% Beta, 1% Delta), and post-second dose (56% WT, 35% Alpha, 28% Beta, 31% Delta). Furthermore, median NAbT against all strains were significantly lower in patients with haematological malignancies vs solid cancers, especially post-second dose (Figure 2c). For both solid and haematological malignancies, the proportion of patients with detectable NAbT and median NAbT were significantly higher in those with previous infection vs infection-naive at baseline, post-first, and post-second vaccine dose, although again values were lower for haematological vs solid cancers (Extended Data Figure 2a,b). Specifically, post-second dose, NAbs against WT were detectable in 80% of patients with haematological malignancy (70% Alpha, 60% Beta, 57% Delta) and 99% of patients with solid cancers (92% Alpha, 80% Beta, 86% Delta).

Patients with haematological malignancies had a range of responses against WT SARS-CoV-2. For example, following two vaccine doses, a higher proportion of patients with multiple myeloma had detectable NAb (WT: 89%, Alpha: 53%, Beta: 21%, Delta: 32%) vs chronic lymphocytic leukaemia (CLL) (WT: 20%, Alpha: 0%, Beta: 10%, Delta: 20%), with accordingly varied median NAbT against WT (multiple myeloma: 131, CLL: <40), though not VOCs (Extended Data Figure 3a). In contrast, in patients with solid cancers, there were no significant differences in NAb response against any variant postsecond dose according to cancer subtype (Extended Data Figure 3b).

### Impact of clinical and treatment characteristics on NAb response

We next used ordinal regression models to assess which patient and cancer characteristics (including systemic anti-cancer therapy [SACT]) associate with NAbT (categorised as undetectable (<40), moderate (40-256), and high (>256)). Considering all patients, lack of previous SARS-CoV-2 infection, AZ vaccine, older age, and haematological malignancy, but not sex or co-morbidities, were associated with reduced NAbT to WT and VOCs both post-first and post-second dose (Supplementary Table 5).

Considering haematological malignancies alone, regression analysis confirmed the previously observed association of haematological malignancy subtype with lower NAbT to WT (but not VOCs) (Supplementary Table 6). Further, anti-CD20 treatment ≤12 months before vaccination was associated with reduced NAbT against WT and VOCs post-first and post-second vaccine dose (Extended Data Figure 4a). There was no significant association between vaccine type and NAbT, but lack of previous infection and older age were significantly associated with reduced NAbT against all variants (Supplementary Table 6).

Considering solid cancers alone, no significant associations with reduced NAbT were found (including cancer subtype and stage, SACT, and disease status after SACT) beyond the lack of previous infection, older age and AZ vaccine (Supplementary Table 7).

Finally, we did not observe any detrimental effects of GCSF, corticosteroids, and immunosuppressive therapy (including in patients with haematological malignancies with active GvHD) on NAbT against any SARS-CoV-2 strains in patients with solid or haematological malignancies (Supplementary Table 6, 7).

### Comparison of NAb responses with individuals without cancer

Next, we compared NAbT induced by vaccination between cancer patients in CAPTURE study, and healthy participants of the Legacy study^27, 28^. Critically, the same neutralising assays were applied in both cohorts. Overall, following two vaccine doses, NAbT against WT were detectable in 100% of healthy Legacy participants (Alpha: 96%, Beta: 86%, Delta: 85%) vs 87% of CAPTURE cancer patients (Alpha: 70%, Beta: 62%, Delta: 63%). Of note, individuals recruited to Legacy were considerably younger and more frequently received the PZ vaccine. We therefore matched CAPTURE and Legacy participants by factors that impact NAbT (see Methods), including vaccine type, previous infection, and age. Due to the heterogeneity between the two cohorts, matching resulted in attrition of individuals available for comparison.

We first assessed infection-naive individuals vaccinated with PZ. The proportion of solid cancer patients (n=49) who had detectable NAbT post-second dose was only numerically lower vs individuals without cancer (n=55) (WT: 98% vs 100%, Alpha: 92% vs 100%, Beta: 86% vs 91%, Delta: 92% vs 95%, respectively) (Extended Data Figure 5a), and the two groups had comparable median NAbT against all variants (Extended Data Figure 5b). However, a significantly lower proportion of patients with haematological malignancies (n=24) vs those without cancer had detectable NAbT (WT: 37%, Alpha: 17%, Beta: 17%, Delta: 17%) (Extended Data Figure 5a), with significantly lower median NAbT against all variants (Extended Data Figure 5b). We note that PZ-vaccinated patients with haematological malignancies were more likely to have CLL or lymphoma, and treatment with anti-CD20 vs AZ-vaccinated patients.

Only a small number of age-matched Legacy participants received AZ vaccine (n=8 infection-naive, age: 40-59 years). Post-second dose, a numerically lower proportion of patients with solid cancers (n=77) had detectable NAbT against all variants other than Delta vs individuals without cancer (WT: 92% vs 100%, Alpha 66% vs 75%, Beta: 60% vs 75%, Delta: 60% vs 50%, respectively) (Extended Data Figure 5c), but this was not statistically significant and median NAbT were comparable (Extended Data Figure 5d). As with PZ, a lower proportion of patients with haematological malignancies (n=18) vs those without cancer had detectable NAbT against all variants (WT: 73%, Alpha: 40%, Beta: 20%, Delta: 36%), with corresponding lower NAbT (Extended Data Figure 5c,d). We note that AZ-vaccinated patients with haematological malignancies were more likely to have ALL or myeloma and less likely to have received anti-CD20 therapy vs PZ-vaccinated patients.

### COVID-19 vaccines induce T-cell responses in cancer patients

**Figure 4:**
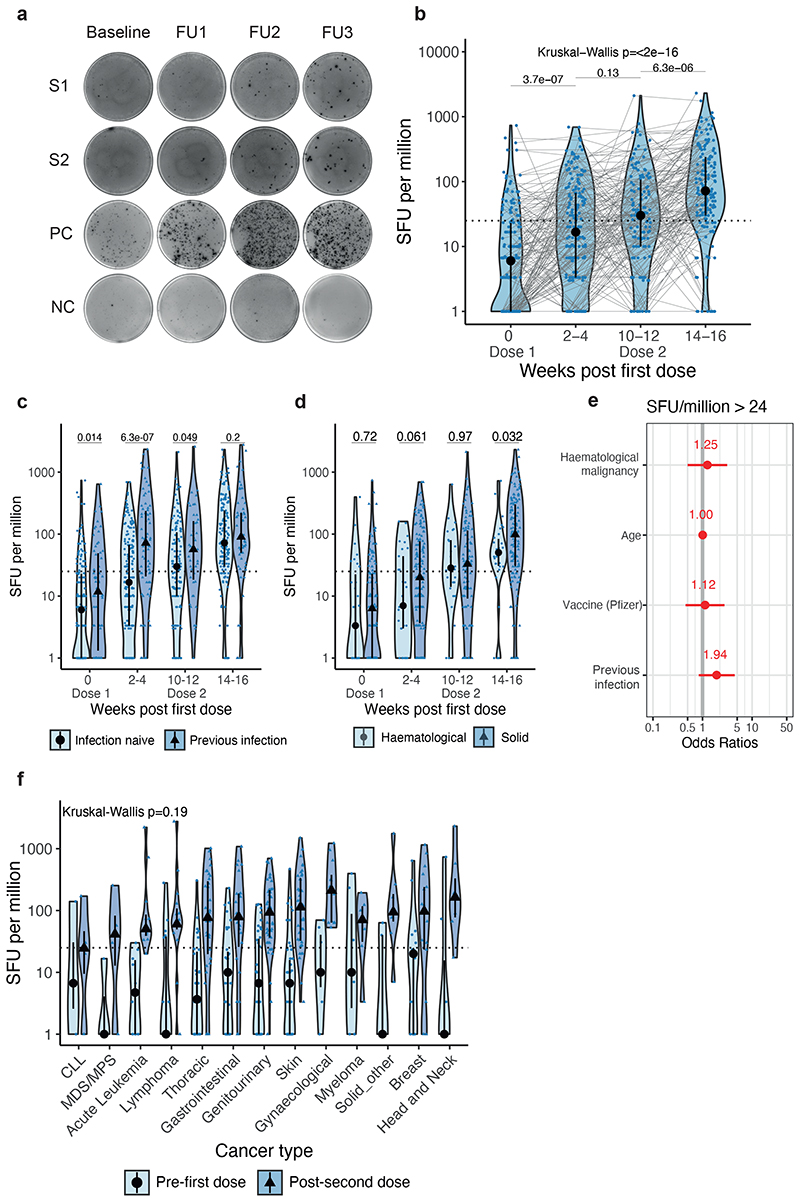
WT SARS-CoV-2-specific T-cell responses in cancer patients following vaccination **a)** Exemplar ELISPOT illustrating WT SARS-CoV-2 specific T-cell response. PBMC were stimulated with 15-mer peptide pools spanning the S1 or S2 subunit of spike. T-cell responses represent the sum of SFU/10^6^ PBMC after stimulation with WT S1 or S2 peptide pools. b) SFU/10^6^ PBMC in infection-naive patients after vaccination (n= 165/195/122/160 patients at BL/FU1/FU2/FU3). Dotted line at <24 denotes the threshold for positivity. Violin plots denote density, PointRange the median and 25 and 75 percentiles. Dots represent individual samples. Samples from individual patients are connected. Significance was tested by Kruskal Wallis test, post-hoc test: two-sided Wilcoxon-Mann-Whitney U test with Bonferroni correction. Only comparisons with the prior timepoint are denoted in the graph. c) Comparison of SFU/10^6^ PBMC in patients with (n= 70/88/49/69 patients at BL/FU1/FU2/FU3) and without prior SARS-CoV-2 infection (n= 165/195/122/160 patients at BL/FU1/FU2/FU3) and in d) patients with solid (n= 136/161/98/130 patients at BL/FU1/FU2/FU3) vs haematological malignancies (n= 29/34/24/30 patients at BL/FU1/FU2/FU3). Violin plots denote density, PointRange the median and 25 and 75 percentiles. Dots represent individual samples. Significance in c-d was tested by two-sided Wilcoxon-Mann-Whitney U test. e) Binary logistic regression of SFU per million PBMCs in patients with solid tumours vs haematological malignancies. Dots denote odds ratio (blue, positive odds ratio red, negative odds ratio); whiskers denote the IQR times 1.5. f) Comparison of SFU per million in patients with haematological malignancies and solid tumours pre-first dose and post-second dose. Dotted line at <24 denotes the lower limit of detection. Violin plots denote density. PointRange denotes the median and the 25 and 75 percentiles. Dots represent individual samples. Significance was tested by Kruskal Wallis test, p < 0.05 was considered significant, post-hoc test: two sided Wilcoxon-Mann-Whitney U test with Bonferroni correction. PBMC, peripheral blood mononuclear cells; NC, negative control; PC, positive control; SFU, spot-forming unit. BL, baseline; FU1, 21-56 days post first-vaccine; FU2, 14-28 days prior to second-vaccine; FU3, 14-28days post second-vaccine.

We evaluated spike-specific T-cell responses following one or two vaccine doses (Supplementary Table 3) by IFN-y ELISPOT after stimulation with WT or Alpha spike peptide pools in a subset of 337 cancer patients (Methods, Figure 4a, Extended Data Figure 1b,c). In 13/337 patients (4%, 10 with solid and 3 with haematological malignancy) all samples were excluded either due to low viable cell count or failed negative or positive control in the assay. Of the 324 remaining patients, 279 had solid (of whom 94% had NAb against WT, 77% Alpha, 73% Beta, 71% Delta), and 58 haematological malignancies (of whom 69% had NAb against WT 2, 49% Alpha, 39% Beta, 45% Delta). Delta spike peptide pools were analysed in a subset of 86 cancer patients.

In the infection-naive patients, T-cell responses to the WT spike peptide pools were detected in 22% of patients at baseline, suggesting cross-reactivity to other human coronaviruses (see also companion paper Fendler et al.). Post-first vaccine dose, 44% of evaluated patients had a detectable T-cell response to the WT spike peptide pools (i.e., >24 spot-forming units [SFU]/10^6^ PBMCs, see Methods), increasing to 56% just prior to second dose, and 79% post-second dose. SFU levels increased significantly both post-first and post-second dose (median 3.3-fold and 13-fold increase vs baseline, respectively; Figure 4b).

Regarding patients with prior SARS-CoV-2 infection, 32% had detectable T-cells responses to the WT spike peptide pools at baseline, increasing to 69% post-first and 87% post-second dose. Median SFU levels to the WT spike were significantly higher vs infection-naive patients at baseline. To confirm increased baseline T-cell responses were related to previous infection, we also measured responses after stimulation with N and M peptide pools and found that median SFU levels were higher at baseline in those with previous infection (Extended Data Figure 6a). Furthermore, we observed a significant increase of SFU levels against Alpha VOC, but not Delta VOC post-second vaccine dose independent of infection status (Extended Data Figure 6b,c).

The proportion of infection-naive patients with haematological malignancies who had T-cell responses to the WT spike peptide pools was only nominally different vs solid cancers (34% vs 45% post-first dose, and 83% vs 78% post-second dose) (Figure 4d). While the SFU levels were significantly lower vs solid cancers post-second dose (median SFU/10^6^ PBMC: 50.5 vs 98.3), in a logistic regression model there was no significant association between detectable T-cell responses and cancer type, patient characteristics or vaccine type (AZ or PZ) (Figure 4e and Extended Data Figure 6d); there was also no significant differences in SFU levels post-second dose between cancer subtypes (Figure 4f). In addition, we detected T-cell responses after two vaccine doses in 4 out of 4 evaluated patients treated with anti-CD20 (Extended Data Figure 6e). Consistent with Th1 cell responses, we detected increased TNF-α, IL-2, IL-18, IL-12 p40, and IP-10 after stimulation with S1/S2 peptide pools vs unstimulated controls (Extended Data Figure 6f), with comparable levels of these cytokines in patients with haematological (n=25) and solid cancers (n=8) (Extended Data Figure 6g). Finally, SFU levels, and the proportion of patients with detectable spike-reactive T-cells (solid: 77%, haematological: 80%) after two vaccine doses were not significantly different to those in healthcare worker controls (80%, n=25) (Supplementary Table 8 and Extended Data Figure 7).

We also observed T-cell responses in patients without detectable NAbs (Supplementary Table 9). For example, in patients with haematological malignancies, T-cell responses were detected in 92% of patients (11/12) without detectable NAbs against WT (in 80% without NAbT against Alpha, 75% Beta, 86% Delta).

### SARS-CoV-2 infection in vaccinated cancer patients

At time of database lock (median 55 days post-second vaccine dose), 1% of patients (8/585; 4 patients AZ, 4 patients PZ) had a positive SARS-CoV-2 RT-PCR, with six patients testing positive between first and second, and two post-second vaccine dose (Extended Data Figure 8). Three patients had a diagnosis of haematological and five patients of solid malignancy. Three patients had evidence of past SARS-CoV-2 infection at the time of first vaccine dose (minimum 30 days since previous positive SARS-CoV-2 RT-PCR test).

Overall, 5/8 patients were identified through routine screening (WHO severity score 1)^30^, of whom four were asymptomatic and one subsequently developed fever and anosmia (WHO score 2). Three patients presented with symptoms (WHO score 3-5) (Extended Data Figure 8).

For technical reasons we were only able to confirm lineage by viral genome sequencing in one patient (Alpha), but given the timing of presentation these patients were likely infected with either Alpha or Delta VOC. At the last evaluable timepoint prior to infection, 6/8 patients had detectable NAb to WT SARS-CoV-2, but fewer had detectable NAbs to VOCs (4/8 Alpha, 4/8 Beta, 4/8 Delta) with correspondingly lower NAbT. The patient with the most severe disease course (CV0217, Extended Data Figure 8) presenting post-first vaccine dose had no evidence of NAb to WT or VOC post-first or post-second vaccine dose or at any time during the course of COVID-19 illness. SARS-CoV-2 specific T-cells were only detectable in 1/4 patients prior to infection and post-first vaccine dose.

## Discussion

Our prospective study of 585 patients with cancer following AZ or PZ COVID-19 vaccination revealed an overall 78% seroconversion rate, with lower rates in haematological (59%) vs solid malignancies (85%). This was numerically comparable to other studies in cancer patients^15–17, 24, 31, 32^, and lower compared with the general population (99%)^33^. Importantly, functionally relevant NAbs against Delta were detectable in only 54% of infection-naive patients with cancer (62% and 31% in solid and haematological malignancies, respectively; 50% and 68% with AZ and PZ, respectively), lower than the reported 85% using the same neutralisation assay, in a younger population without cancer^27, 28^.

Given the complete dominance of Delta in the UK and surging prevalence globally, our data on NAb activity against VOCs have contemporary implications for the care of cancer patients who are at increased risk of adverse outcomes of SARS-CoV-2 infection. Studies in cancer patients to date have used seroconversion (i.e., detection of IgG antibodies against WT spike) as the main immunogenicity endpoint^14–24, 31^, but NAb against VOCs have not been evaluated. Although we found good concordance between the presence of anti-S1 IgG antibodies and NAbT against WT SARS-CoV-2 in our cohort (in line with reports in those without cancer)^34–36^, seroconversion was a poorer surrogate for NAbT against VOCs, where approximately half the patients without detectable NAb against Delta had anti-S1 IgG antibodies. The recombinant S1 protein used in the serological assay corresponds to the WT sequence, and selection of spike mutations in the VOCs leads to diminished neutralising activity of such antibodies. Given that NAb are highly predictive of immune protection from symptomatic SARS-CoV-2 infection ^34, 37, 38^, our data suggest that serological assays may underestimate the risk of breakthrough infection, when not accounting for viral evolution and the disconnect with NAbT against VOCs.

An inverse relationship between age and vaccine-induced neutralising responses was recently shown in non-cancer subjects, with those aged >80 years particularly affected^39^. Likewise, in our cohort of patients with cancer, increasing age correlated with reduced NAbT. The unmatched comparison of CAPTURE cancer patients with the younger Legacy cohort (median age 35.3 years) also showed reduced NAb, further highlighting the effect of age on vaccine response. Given the relatively young median age in our cohort (60 years), it is possible that the effect of age in the general cancer population is even more pronounced.

The mix of patients who received AZ or PZ vaccines, delivered 12 weeks apart as per current UK guidelines, uniquely facilitated assessment of differential responses to the two vaccines within a lengthened timeframe. Despite maintained seroconversion rates between doses for either vaccine, the interval between first and second dose still represents an ‘at-risk’ period, where neither vaccine led to a robust NAb response against VOCs. Post-first dose, NAbT against Delta were undetectable for 90% (AZ) and 95% (PZ) of patients, though NAbT against WT was higher with AZ vs PZ. Post-second dose, NAbT increased but the levels were still diminished against VOCs vs WT. This was more pronounced with AZ vs PZ (50% vs 68% of infection-naive patients had detectable NAbT against Delta after two doses), consistent with the modestly reduced effectiveness of AZ (67%) vs PZ (88%) against Delta VOC in the general UK population ^40^. The implications of our findings are two-fold. First, a proportion of patients with cancer who are ‘double-vaccinated’ may still be suboptimally protected when transmission rates of VOCs in the community are high. Second, while broad debate remains on optimal dosing schedule of two-dose regimens (by efficacy or resource distribution arguments), our data suggest a shorter interval (<12 weeks) between vaccine doses may minimise the ‘at risk’ period for cancer patients who do not develop NAbs during the prolonged dosing schedule. A potential tradeoff to this may be overall lower antibody titres with a shortened schedule ^41, 42^, but this may conceivably be rescued with a third vaccine dose.

We note that the differences in NAbT between AZ and PZ in our cohort, consistent with the findings in patients on haemodialysis^29^, are largely driven by patients with solid cancers. In patients with haematological malignancies, NAb responses were generally low, without a discernible impact of the vaccine type. NAbT were lowest in patients treated with anti-CD20 antibodies, and patients with CLL were more likely to lack Nab than those with multiple myeloma (Delta: 0% vs 32%, respectively). Irrespective of underlying malignancy type, NAbT against VOCs were augmented by prior SARS-CoV-2 infection with incremental increase in seroconversion and NAbT following two vaccine doses. This suggests that patients with cancer, especially those with haematological malignancies, would benefit from a third vaccine dose to further boost humoral immunity. Two recent studies in solid-organ transplant recipients (n=101^43^ and n=120^44^), where the third dose significantly improved immunogenicity of the PZ vaccine, lend further support to this notion (although the differences between infection and vaccination in antigen load and degree of T-/B-cell stimulation need to be acknowledged). Furthermore, recent data on the added benefits of heterologous vaccination regimens^45–48^, through boosting of both antibody and T-cell responses, may be especially relevant for patients with haematological malignancies who have lower NAb responses to both AZ and PZ. We also note a report of a patient with lymphoplasmacytic lymphoma treated with rituximab (anti-CD20) who failed to seroconvert after two doses of the PZ vaccine, but developed NAbs following a booster with JNJ-78436735 (Johnson&Johnson, a viral vector vaccine)^49^. Prospective data are needed to determine the optimal vaccination regimen in immunocompromised patients.

In the most substantial evaluation of cellular immunity to COVID-19 vaccines in cancer patients to date (N=324), we observed SARS-CoV-2-specific T-cell responses in the majority of patients, and responses were in a range similar to that of healthy individuals. Importantly, we detected T-cell responses against Alpha and Delta peptide pools, in agreement with a recent report suggesting that T-cells induced by the WT SARS-CoV-2 were effective against VOCs ^50^. Critically, in our cohort, T-cell responses were observed in most patients with haematological malignancies, including those with undetectable NAbT. Additionally, patients with solid and haematological malignancies had comparable Th1-driven responses. The dissonance of humoral and cellular responses was also seen with SARS-CoV-2 infection (companion paper Fendler *et al*.;^51^), including in patients on anti-CD20 therapy, suggesting cellular immunity offers some immune protection in this patient group. Overall, however, our understanding of T-cell role in immune protection from SARS-CoV-2 remains incomplete; while they are not expected to prevent infection, T-cell responses are likely to reduce COVID-19 severity. Pre-clinical studies in mice^52^ and rhesus macaques^53^ have demonstrated the role of cellular immunity in SARS-CoV-2 clearance. A study in patients with multiple sclerosis on anti-CD20 treatment (n=20) reported suppressed humoral responses but augmented CD8 T-cell induction and preserved Th1 priming following COVID-19 vaccination^54^. Overall, the absolute excess risk for postvaccination breakthrough infection with skewed immunity towards a cellular response is unquantified.

Among patients with solid malignancy, cancer subtype did not impact neither NAbT nor T-cell responses to vaccination. Noteworthy, in patients with thoracic malignancies, who are known to be at higher risk of severe outcomes to COVID-19 ^8, 11, 55, 56^, vaccine-induced immunity was not inferior vs other solid cancers. Furthermore, systemic therapy, including CPI and corticosteroids were not detrimental to induction of immune response to vaccination. This is reassuring and further reflected by the finding that median NAbT among PZ-vaccinated patients with solid cancers were comparable to that in age-matched individuals without cancer from the Legacy cohort^27, 28^, though the conclusions for AZ are more limited by the very small sample size. While a relatively small number of patients with solid cancer had undetectable NAb against Delta (AZ: 36%, PZ: 8%), the proportion was overall higher vs healthy controls. Our study is underpowered to definitively ascertain whether cancer-specific factors impact NAb response in patients with solid cancer, or if this is largely driven by age. While our data in this patient group are overall reassuring, it is important to acknowledge that NAb levels required to prevent infection may be higher than those needed for prevention of severe illness. Prevention of SARS-CoV-2 infection in cancer patients, especially those in active treatment, is critical, as even asymptomatic infections can interrupt delivery of cancer care (i.e. surgery, SACT, hospital appointments).

We observed only eight breakthrough SARS-CoV-2 infections (1% of vaccinated patients). However, the study period fell between February and May 2021 for most patients, representing a time of relatively low infection rates in the UK, among declining Alpha VOC infections and prior to the current Delta surge. During a similar time period (December 2020 - May 2021), a longitudinal community surveillance study of the general UK population showed that vaccination with one dose of AZ/PZ reduced infections by 61%-66% (further reduction by 79%-80% with second dose)^57^. Our low rate of breakthrough infections in cancer patients is reassuring, but as CAPTURE was not designed to assess vaccine efficacy this needs to be considered with caution. Further, behaviour of cancer patients may have contributed, as they are likely to exercise caution especially prior to full vaccination. An ongoing aim of the CAPTURE study includes collection of data on breakthrough infections.

The strengths of our study include a large, prospectively recruited cohort with comparison across humoral and cell mediated immunity against VOCs, which has so far been lacking in studies of patients with cancer. There are limitations in our dataset; firstly, while we performed an age-matched comparison with the Legacy data, the analysis was limited to a small number of patients and would benefit from further validation. Secondly, we relied on opportunistic sampling given restrictions on non-essential travel and hospital attendance leading to missed sampling points, particularly in occasional hospital attendees. Finally, validation of findings in solid cancer type/treatment subgroups in larger datasets, or through meta-analyses will be important especially for detection of marginal differences.

In conclusion, our results have clear implications for the management of patients with cancer. Our data support the prioritisation of patients with cancer for booster vaccine doses, suggesting that highest priority should be given those with haematological malignancies, followed by patients with advanced age, especially if vaccinated with AZ. Personal risk mitigation and ongoing public health measures remain relevant, for the at-risk groups, especially when community transmission of VOCs is high. Moving forward, defining the correlates of immune protection (including humoral and cellular responses) will be critical to guide decision making. Longitudinal evaluation will define the durability and nature of immune protection and the occurrence of breakthrough infection in the context of potentially waning antibody responses. As such, an adaptable framework within ongoing prospective efforts will be instrumental to safely navigate the next phase of the pandemic for our patients.

## Methods

### Study design

CAPTURE (NCT03226886) is a prospective, longitudinal cohort study that commenced recruitment in May 2020, and continues to enrol patients at the Royal Marsden NHS Foundation Trust. The study design has been previously published^26^. In brief, adult patients with current or history of invasive cancer are eligible for enrolment. Inclusion criteria are intentionally broad, and patients are recruited irrespective of cancer type, stage, or treatment. Patients recruited to the CAPTURE study who have received at least one dose of COVID-19 vaccine will be included in an analysis to explore vaccine immunogenicity in cancer patients. Patients are included in the analysis regardless of prior SARS-CoV-2 infection status. The primary outcome for this analysis will be the seroconversion rate in cancer patients at 14-28 days following the second dose of vaccine. At establishment of the study protocol, there was no prior published data of seroconversion in cancer patients in this setting and thus sample size was exploratory. The most precise estimate of seroconversion in cancer patients would therefore be achieved through recruitment of as many patients as possible in the time period.

CAPTURE was approved as a substudy of TRACERx Renal (NCT03226886). TRACERx Renal was initially approved by the NRES Committee London, Fulham, on January 17, 2012. The TRACERx Renal sub-study CAPTURE was submitted as part of Substantial Amendment 9 and approved by the Health Research Authority on April 30, 2020 and the NRES Committee London - Fulham on May 1, 2020. CAPTURE is being conducted in accordance with the ethical principles of the Declaration of Helsinki, Good Clinical Practice and applicable regulatory requirements. All patients provided written, informed consent to participate.

### Study schedule and follow-up

Clinical data and sample collection for participating cancer patients is performed at baseline (pre-first dose vaccine or within 14 days of first dose vaccine), at timepoints follow-up 1 (FU1; 2-4 weeks post-first dose vaccine); FU2 (within 14 days prior to second vaccine); FU3 (2-4 weeks post-second dose vaccine) (see Figure 1a and Supplementary Material Study Protocol).

### Patient data and sample Sources

Demographic, epidemiological and clinical data (e.g. cancer type, cancer stage, treatment history) were collected from the internal electronic patient record and pseudonymised data was entered into in a cloud-based electronic database (Ninox Software, Berlin, Germany). Regarding SACT, we deemed chemotherapy, targeted therapy (small molecule inhibitors or monoclonal antibodies) or endocrine therapy to be current if given within 28 days of vaccination. CPI given within six months was considered significant given the prolonged receptor occupancy with these agents^58^. Concomitant medications were recorded for corticosteroids (considered significant if >10mg prednisolone equivalent given for at least 7 days); GCSF when given within 48 hours of vaccination or five days if pegylated preparation; other immunosuppressive drugs taken within 48 hours of vaccination.

Patients were grouped by cancer diagnosis (solid vs hematological malignancy) for downstream analysis. Where two independent diagnoses of cancer were identified in the same patient, the case was reviewed by two clinicians (STCS & AMS) and the highest stage and/or cancer receiving active treatment was used for classification. Solid cancers were subdivided by anatomical systems (Table 1) with 21 patients assigned to the ‘solid other’ category consisting of endocrine and neuroendocrine tumours, sarcoma and gastrointestinal stromal tumours and central nervous system tumours. Patients with haematological malignancies were grouped by conventional subtypes although one patient with aplastic anaemia (CV0611) was not possible to intuitively group with other haematological disorders and excluded from subgroup analyses.

Detailed sampling schedule and methodology has been previously described^26^. Study biospecimens included per-protocol blood samples, oropharyngeal swabs and cryostored serum from routine clinical investigations. Collected data and study samples are de-identified and stored with only the study-specific study identification number.

### Comparison with healthy individuals

Healthy individuals were included from the previous published Legacy study for comparison ^27, 28^. The Legacy study includes healthy individuals vaccinated with PZ or AZ. To account for the heterogeneity of both cohorts, we selected cases based on age, type of vaccine, and infection status. We only included blood samples taken between 14 and 42 days post second dose. Infection status was self-reported within Legacy ^27, 28^. For individuals vaccinated with PZ, we only considered infection naive individuals. Cancer patients and healthy controls were grouped into two age groups for comparison (40-54 years, 55 years and over). Individuals vaccinated with AZ were compared to cancer patients independent of previous infection and only individuals between 40 and 59 years were selected for comparison. T-cell responses were compared to a group of healthcare professionals recruited to the CAPTURE study (n=25, Supplementary Table 8).

### Definition of previous SARS-CoV-2 infection

Most patients underwent RT-PCR screening as part of routine clinical care. To account for asymptomatic infections and/or symptomatic infections not confirmed by RT-PCR, we considered patients to have had previous SARS-CoV-2 infection if they had either i) previous SARS-CoV-2 positive RT-PCR and/or ii) positive anti-S1 IgG ELISA prior to vaccination.

### WHO classification of severity of COVID-19

We classified severity of COVID-19 according to the WHO clinical progression scale^30^. Uninfected: uninfected, no viral RNA detected - 0; Asymptomatic: viral RNA and/or S1-reactive IgG detected – 1; mild (ambulatory): symptomatic, independent – 2; symptomatic, assistance needed - 3; moderate (hospitalised): no oxygen therapy (if hospitalised for isolation only, record status as for ambulatory patient) – 4; oxygen by mask or nasal prongs - 5; severe (hospitalised): oxygen by non-invasive ventilation or high flow – 6; intubation and mechanical ventilation, pO_2_/FiO_2_ ≥ 150 or SpO_2_/FiO_2_ ≥ 200 – 7; mechanical ventilation, pO_2_/FiO_2_ < 150 (SpO_2_/FiO_2_ < 200) or vasopressors – 8; mechanical ventilation, pO_2_/FiO_2_ < 150 and vasopressors, dialysis, or extracorporeal membrane oxygenation - 9; Dead - 10.

### Handling of whole blood samples

All blood samples and isolated products were handled in a CL2 laboratory inside a biosafety cabinet using appropriate personal protective equipment and safety measures, which were in accordance with a risk assessment and standard operating procedure approved by the safety, health and sustainability committee of the Francis Crick Institute. For indicated experiments, serum or plasma samples were heat-inactivated at 56°C for 30 minutes prior to use after which they were used in a CL1 laboratory.

### Plasma and PBMC isolation

Whole blood was collected in EDTA tubes (VWR) and stored at 4°C until processing. All samples were processed within 24 hours. Time of blood draw, processing, and freezing was recorded for each sample. Prior to processing tubes were brought to room temperature (RT). PBMC and plasma were isolated by density-gradient centrifugation using pre-filled centrifugation tubes (pluriSelect). Up to 30 ml of undiluted blood was added on top of the sponge and centrifuged for 30 minutes at 1000g at RT. Plasma was carefully removed then centrifuged for 10 minutes at 4000g to remove debris, aliquoted and stored at -80°C. The cell layer was then collected and washed twice in PBS by centrifugation for 10 minutes at 300g at RT. PBMC were resuspended in Recovery cell culture freezing medium (Fisher Scientific) containing 10% DMSO, placed overnight in CoolCell freezing containers (Corning) at -80°C and then stored at -80°C.

### Serum isolation

Whole blood was collected in serum coagulation tubes (Vacuette CAT tubes, Greiner) for serum isolation and stored at 4°C until processing. All samples were processed within 24 hrs. Time of blood draw, processing, and freezing was recorded for each sample. Tubes were centrifuged for 10 minutes at 2000g at 4°C. Serum was separated from the clotted portion, aliquoted and stored at -80°C.

### S1-reactive IgG ELISA

Ninety-six-well MaxiSorp plates (Thermo Fisher Scientific) were coated overnight at 4°C with purified S1 protein in PBS (3 μg/ml per well in 50 μl) and blocked for 1 hour in blocking buffer (PBS, 5% milk, 0.05% Tween 20, and 0.01% sodium azide). Sera were diluted in blocking buffer (1:50). Fifty μl of serum were then added to the wells and incubated for 2 hours at RT. After washing four times with PBS-T (PBS, 0.05% Tween 20), plates were incubated with alkaline phosphatase-conjugated goat antihuman IgG (1:1000, Jackson ImmunoResearch) for 1 hour. Plates were developed by adding 50 μl alkaline phosphatase substrate (Sigma Aldrich) for 15-30 minutes after six washes with PBS-T. Optical densities were measured at 405 nm on a microplate reader (Tecan). CR3022 (Absolute Antibodies) was used as a positive control. The cut-off for a positive response was defined as the mean negative value multiplied by 0.35 times the mean positive value.

### Virus variants & culture

The SARS-CoV-2 reference isolate (referred to as ‘Wild-type’) was hCoV19/England/02/2020, obtained from the Respiratory Virus Unit, Public Health England, UK, (GISAID EpiCov accession EPI_ISL_407073). The B.1.1 strain (“D614G”) was isolated from a swab from an infected healthcare worker at UCLH, obtained through the SAFER study,2 and carries only the D614G mutation in its spike. The B.1.1.7 isolate (“B.1.1.7”) was the hCoV19/England/204690005/2020, which carries the D614G, Δ69-70, Δ144, N501Y, A570D, P681H, T716I, S982A and D1118H mutations,3 obtained from Public Health England (PHE), UK, through Prof. Wendy Barclay, Imperial College London, London, UK through the Genotype-to-Phenotype National Virology Consortium (G2P-UK). The B.1.351 virus isolate was the 501Y.V2.HV001, which carries the D614G, L18F, D80A, D215G, Δ242-244, K417N, E484K, N501Y, A701V mutations, and was kindly provided by Prof. Alex Sigal and Prof. Tulio de Oliveira; 4 sequencing of viral isolates received identified the Q677H and R682W mutations at the furin cleavage site in approximately 50% of the genomes, which was maintained upon passage in cell culture. The B.1.617.2 isolate was MS066352H (GISAID accession number EPI_ISL_1731019), which carries the T19R, K77R, G142D, Δ156-157/R158G, A222V, L452R, T478K, D614G, P681R, D950N, and was kindly provided by Prof. Wendy Barclay, Imperial College London, London, UK through the Genotype-to-Phenotype National Virology Consortium (G2P-UK). All viral isolates were propagated in Vero V1 cells. Briefly, 50% confluent monolayers of Vero E6 cells were infected with the given SARS CoV-2 strains at an MOI of approx. 0.001. Cells were washed once with DMEM (Sigma; D6429), then 5 ml virus inoculum made up in DMEM was added to each T175 flask and incubated at room temperature for 30 minutes. DMEM + 1% FCS (Biosera; FB-1001/500) was added to each flask. Cells were incubated at 37° C, 5% CO^2^ for 4 days until extensive cytopathogenic effect was observed. Supernatant was harvested and clarified by centrifugation at 2000 rpm for 10 minutes in a benchtop centrifuge. Supernatant was aliquoted and frozen at -80°C.

### Virus PCR and sequencing

All virus stocks generated for use in neutralisation assays were sequence-validated prior to use. To confirm the identity of cultured VoC samples, 8ul of viral RNA was prepared for sequencing by the ARTIC method (https://www.protocols.io/view/ncov-2019-sequencingprotocol-v3-locost-bh42j8ye) and sequenced on the ONT GridION platform to >30k reads / sample. The data was demultiplexed and processed using the viralrecon pipeline (https://github.com/nf-core/viralrecon).

### High-throughput live virus microneutralisation assay

High-throughput live virus microneutralisation assays were performed as described previously^59^. Briefly, Vero E6 cells (Institut Pasteur) or Vero E6 cells expressing ACE2 and TMPRSS2 (VAT-1) (Centre for Virus Research)^60^ at 90-100% confluency in 384-well format were first titrated with varying MOIs of each SARS-CoV-2 variant and varying concentrations of a control monoclonal nanobody in order to normalise for possible replicative differences between variants and select conditions equivalent to wild-type virus. Following this calibration, cells were infected in the presence of serial dilutions of patient serum samples. After infection (24 hrs Vero E6 Pasteur, 16hrs VAT-1), cells were fixed with 4% final Formaldehyde, permeabilised with 0.2% TritonX-100, 3% BSA in PBS (v/v), and stained for SARS-CoV-2 N protein using Alexa488-labelled-CR3009 antibody produced in-house and cellular DNA using DAPI^61^. Whole-well imaging at 5x was carried out using an Opera Phenix (Perkin Elmer) and fluorescent areas and intensity calculated using the Phenix-associated software Harmony 9 (Perkin Elmer). Inhibition was estimated from the measured area of infected cells/total area occupied by all cells. The inhibitory profile of each serum sample was estimated by fitting a 4-parameter dose response curve executed in SciPy. Neutralising antibody titres are reported as the fold-dilution of serum required to inhibit 50% of viral replication (IC50), and are further annotated if they lie above the quantitative (complete inhibition) range, below the quantitative range but still within the qualitative range (i.e. partial inhibition is observed but a dose- response curve cannot be fit because it does not sufficiently span the IC50), or if they show no inhibition at all. IC50 values above the quantitative limit of detection of the assay (>2560) were recoded as 3000; IC50 values below the quantitative limit of the assay (< 40) but within the qualitative range were recoded as 39 and data below the qualitative range (i.e. no response observed) were recoded as 35.

### ELISpot assay

IFN-γ Precoated ELISpot (Mabtech, UK) plates were blocked with complete medium (RPMI, 5% human AB serum) before 300,000 PBMC were seeded per well and stimulated for 18 h. Synthetic SARS-CoV-2 PepTivator peptides (Miltenyi Biotec, Surrey, UK), consisting of 15-mer sequences with 11 amino acid overlap were used at a final concentration of 1 μg/ml/peptide, as follows: (1) PepTivator SARS-CoV-2 Prot_S1 (amino acids 1-692); (2) PepTivator SARS-CoV-2 Prot_S (covering the sequences 304-338, 421-475, 492-519, 683-707, 741-770, 785-802 and 885-1273) and PepTivator SARS-CoV-2 Prot_S+ (amino acids 689-895) combined into a single pool broadly representing S2; (3) PepTivator SARS-CoV-2 Prot_M (covering the complete membrane glycoprotein); (4) PepTivator SARS-CoV-2 Prot_N (covering the complete nucleocapsid phosphoprotein), (5) PepTivator SARS-CoV-2 Prot_S B.1.1.7 Mutation Pool (34 peptides covering the mutated regions in spike of the Alpha VOC); (6) The PepTivator SARS-CoV-2 Prot_S B.1.617.2 Mutation Pool covers selectively the mutated regions (32 peptides covering the mutated regions in spike of the Delta).

Plates were developed with human biotinylated IFN-γ detection antibody (7-B6-1-ALP, 1:200), followed by incubation BCIP/NBT Phosphatase Substrate (SeraCare). Spot forming units (Mabtech) were quantified with ImmunoSpot. To quantify positive peptide-specific responses, spots of the unstimulated wells were subtracted from the peptide-stimulated wells, and the results expressed as SFU per million. Samples where positive controls were <10 SFU/10^6^ spots per well were excluded, as were samples with negative control >50 SFU/10^6^. The cut-off threshold for a positive result was the mean of the negative control well plus 2 times the standard deviation (24 SFU/10^6^)^62^. The magnitude of the response (ie, SFU/10^6^) could not be compared between WT and VOCs due to the reduced number of peptides in the VOC pools.

### Multiplex immune assay for cytokines and chemokines

The Milliplex Human Cytokine Panel A immunoassay (Merck, UK) was used to measure 15 protein targets in cell culture supernatants on the Bio-Plex platform (Bio-Rad Laboratories, Hercules, CA, USA), using Luminex xMAP technology. Analytes measured included: IFN-y, IL-10, IL-12p40, IL-12p70, IL-13, IL-17A, IL-18, IL-2, IL-22, IL-4, IL-5, IL-9, IP-10, MCP-1, TNF-A. All assays were conducted as per the manufacturer’s recommendation.

### Quantification and statistical analysis

Data and statistical analysis were done in R v3.6.1 in R studio v1.2.1335. Gaussian distribution was tested by Kolmogorov-Smirnov test. Wilcoxon-Mann-Whitney, Kruskal-Wallis, Chi2, Fisher’s exact test were performed for statistical significance. Bonferroni correction was applied for multiple-comparison testing. A p-value <0.05 was considered significant. All tests were performed two-sided. Statistical details for each experiment are provided in the figure legends. The ggplot2 package in R was used for data visualization and illustrative figures were created with BioRender.com. Data are usually plotted as single data points and violin or box plots on a logarithmic scale. For boxplots, boxes represent upper and lower quartiles, line represents median, and whiskers IQR times 1.5. Notches represent confidence intervals of the median. PointRange in violin plots denotes median and upper and lower quartiles. Spearman rank correlation coefficients were calculated between all parameter pairs. Multivariate binary logistic regression analysis was performed using the glm function with the stats package in R. Ordinal logistic regression was performed using the orm function with the rms package in R.

## Extended Data

**Figure:**
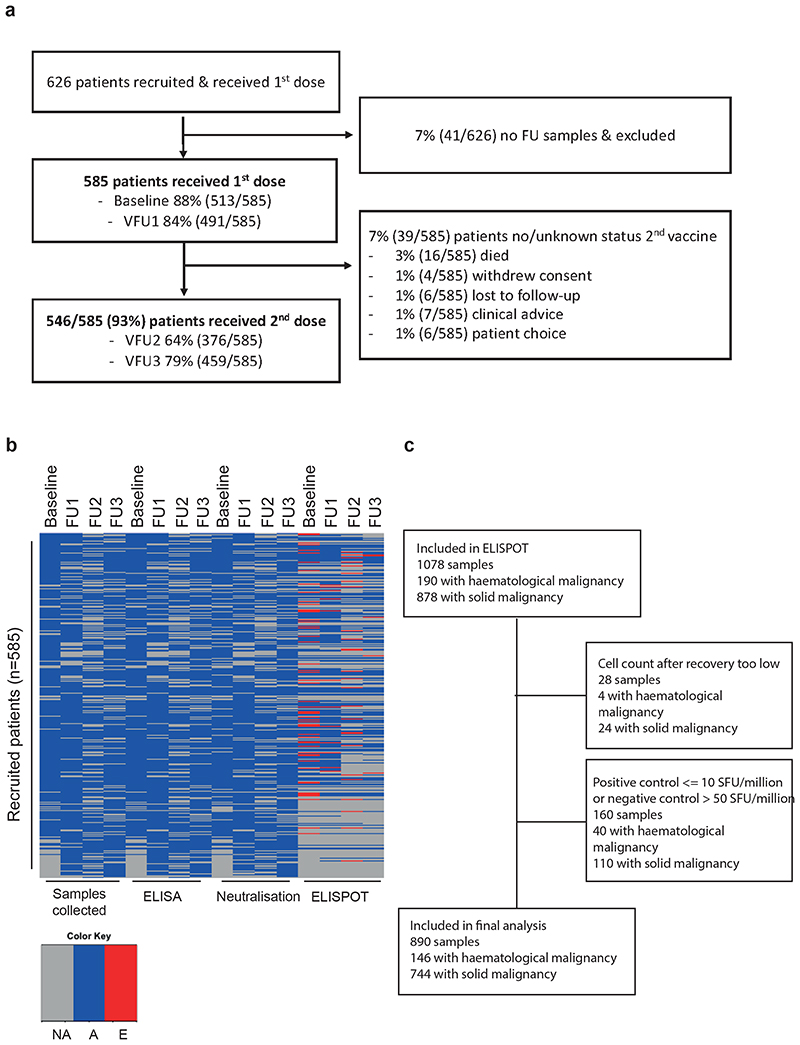


**Figure:**
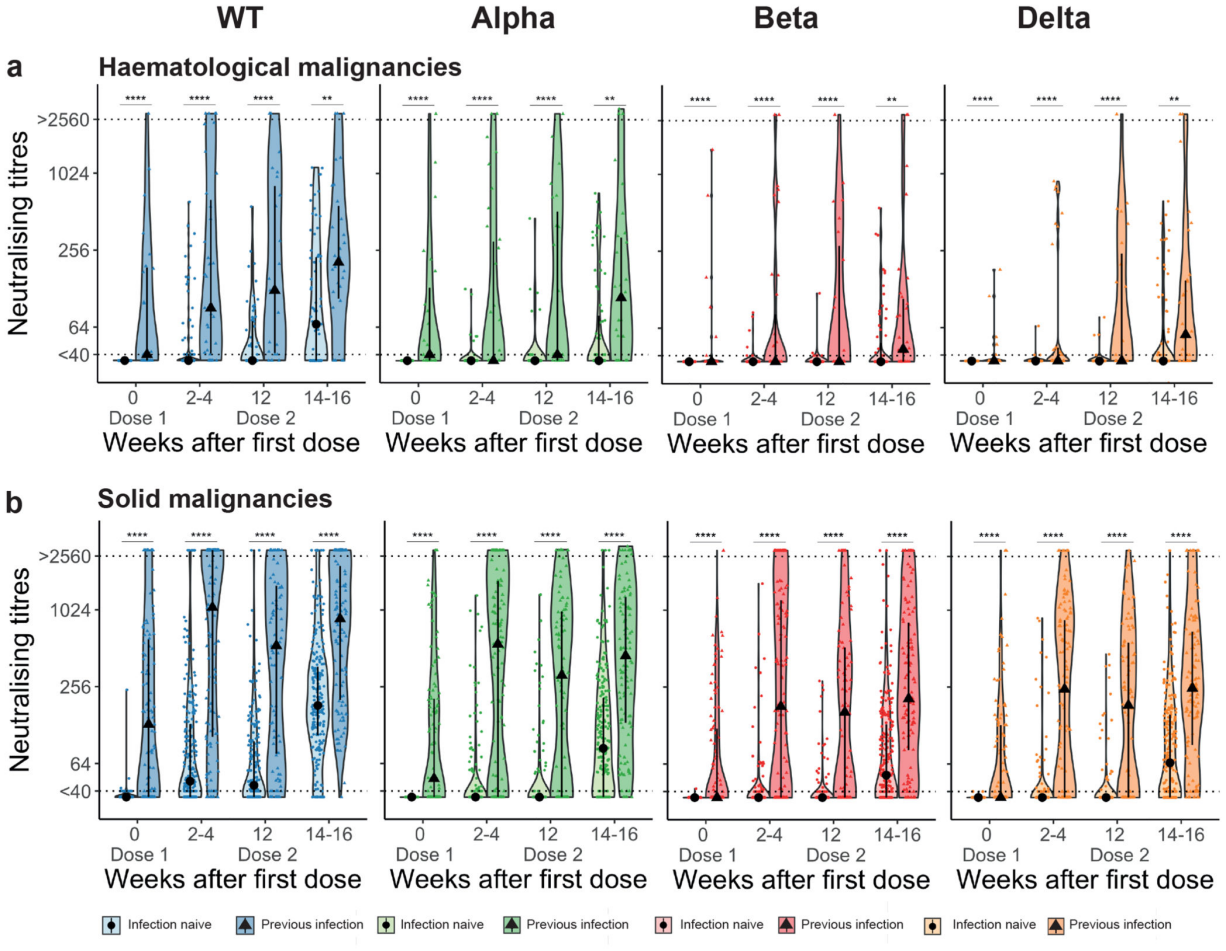


**Figure:**
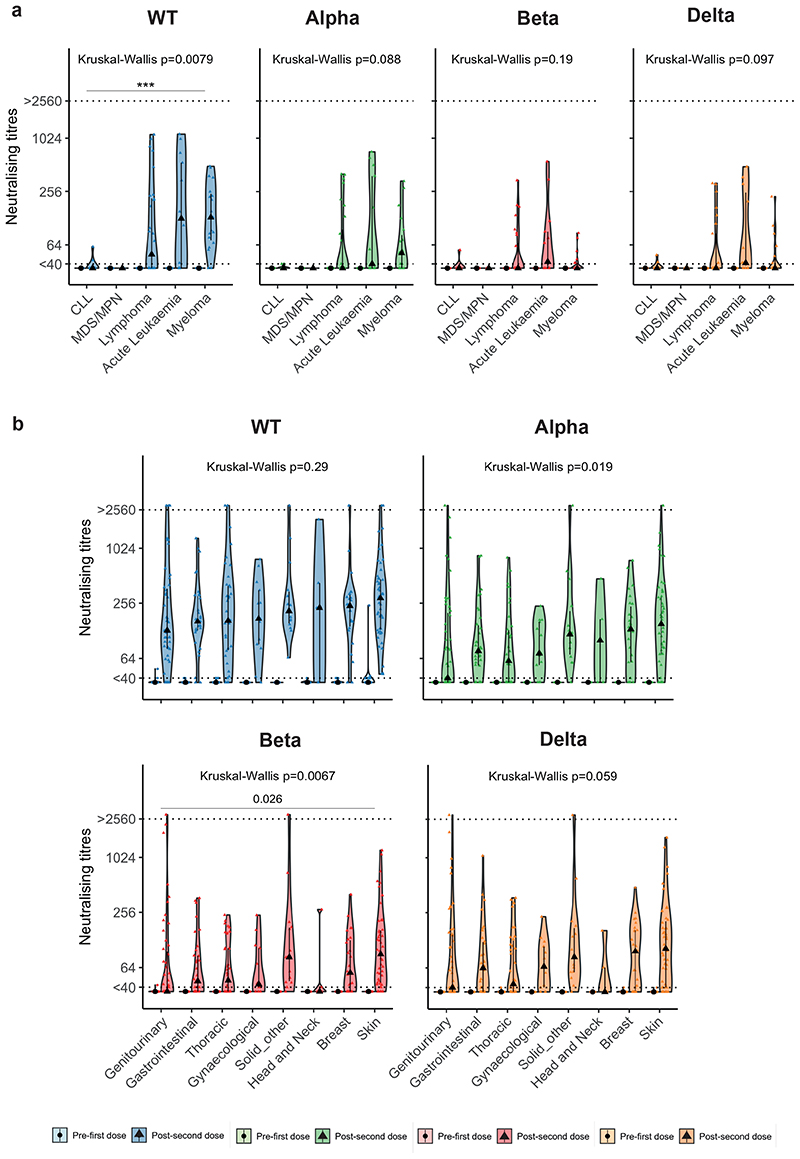


**Figure:**
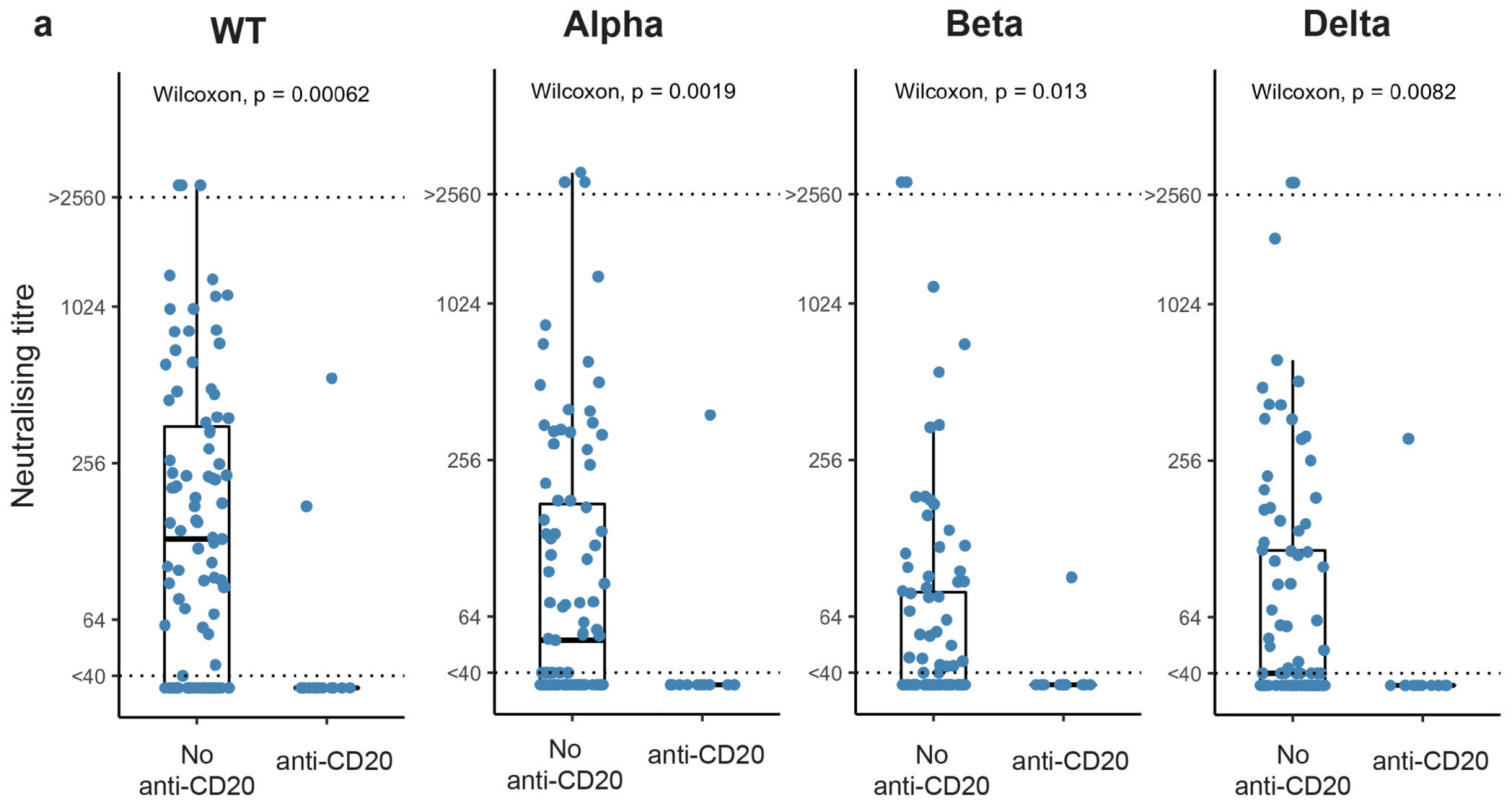


**Figure:**
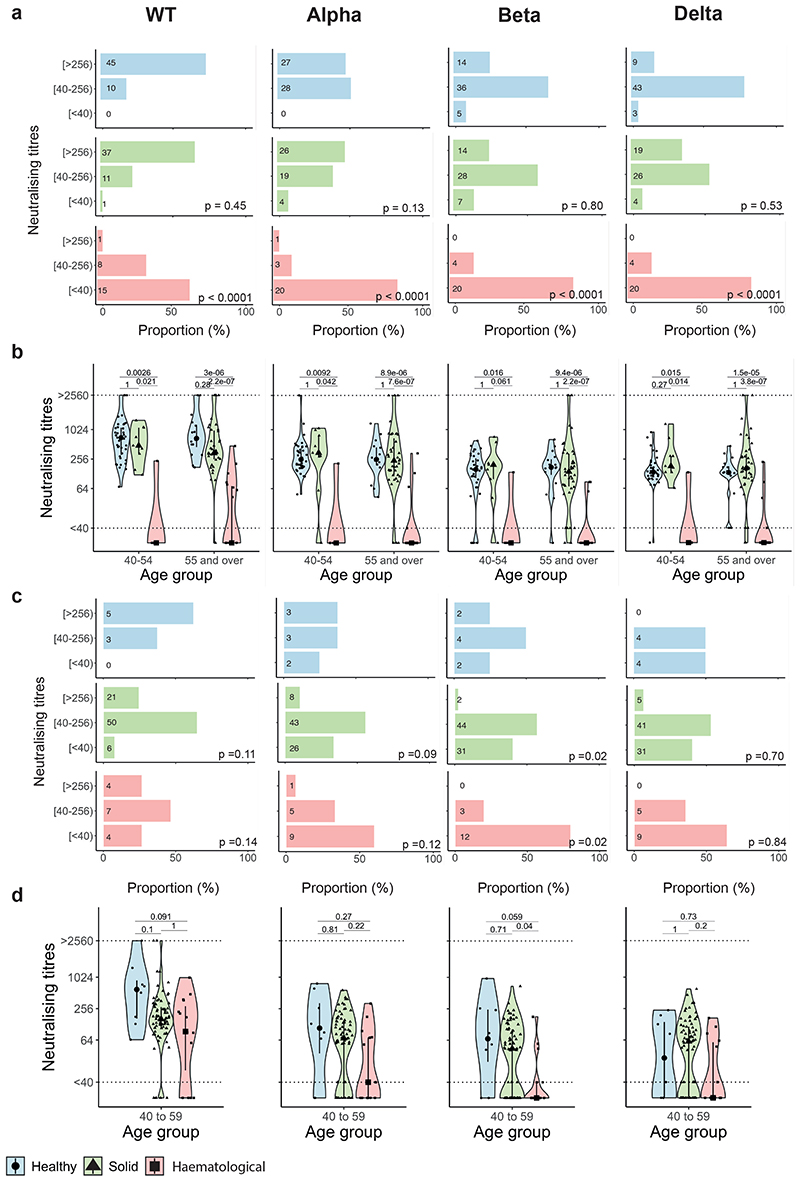


**Figure:**
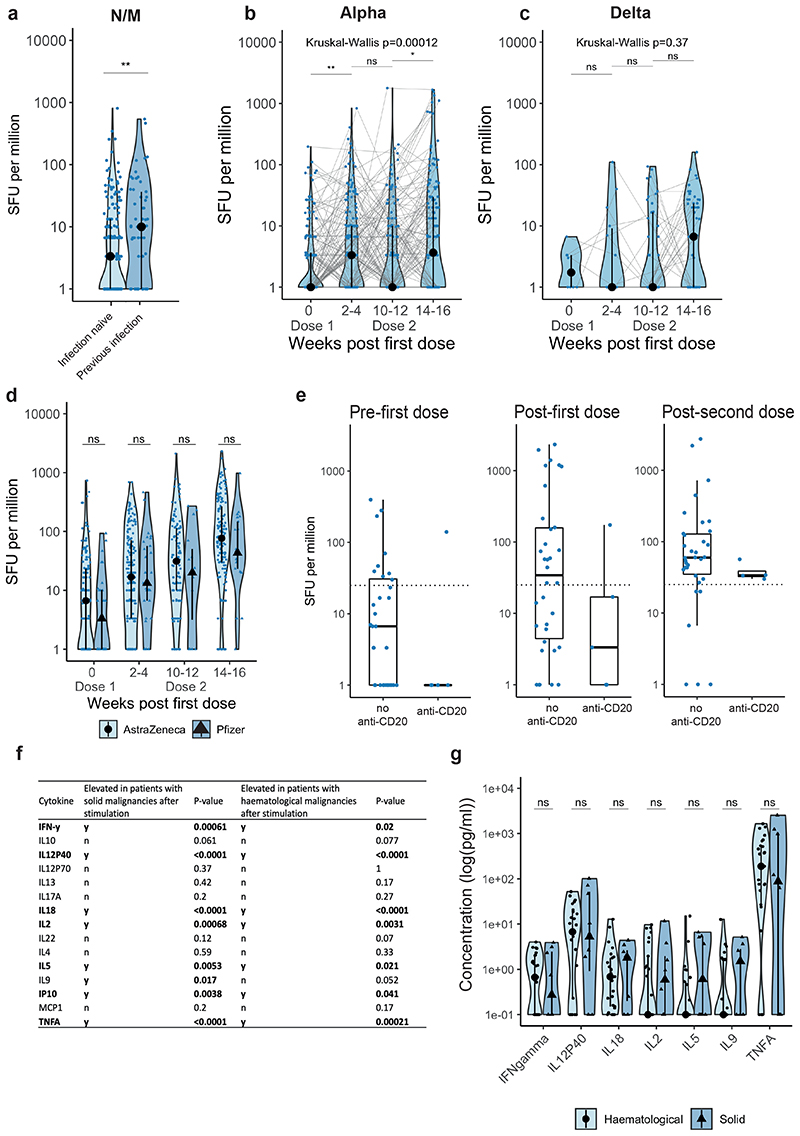


**Figure:**
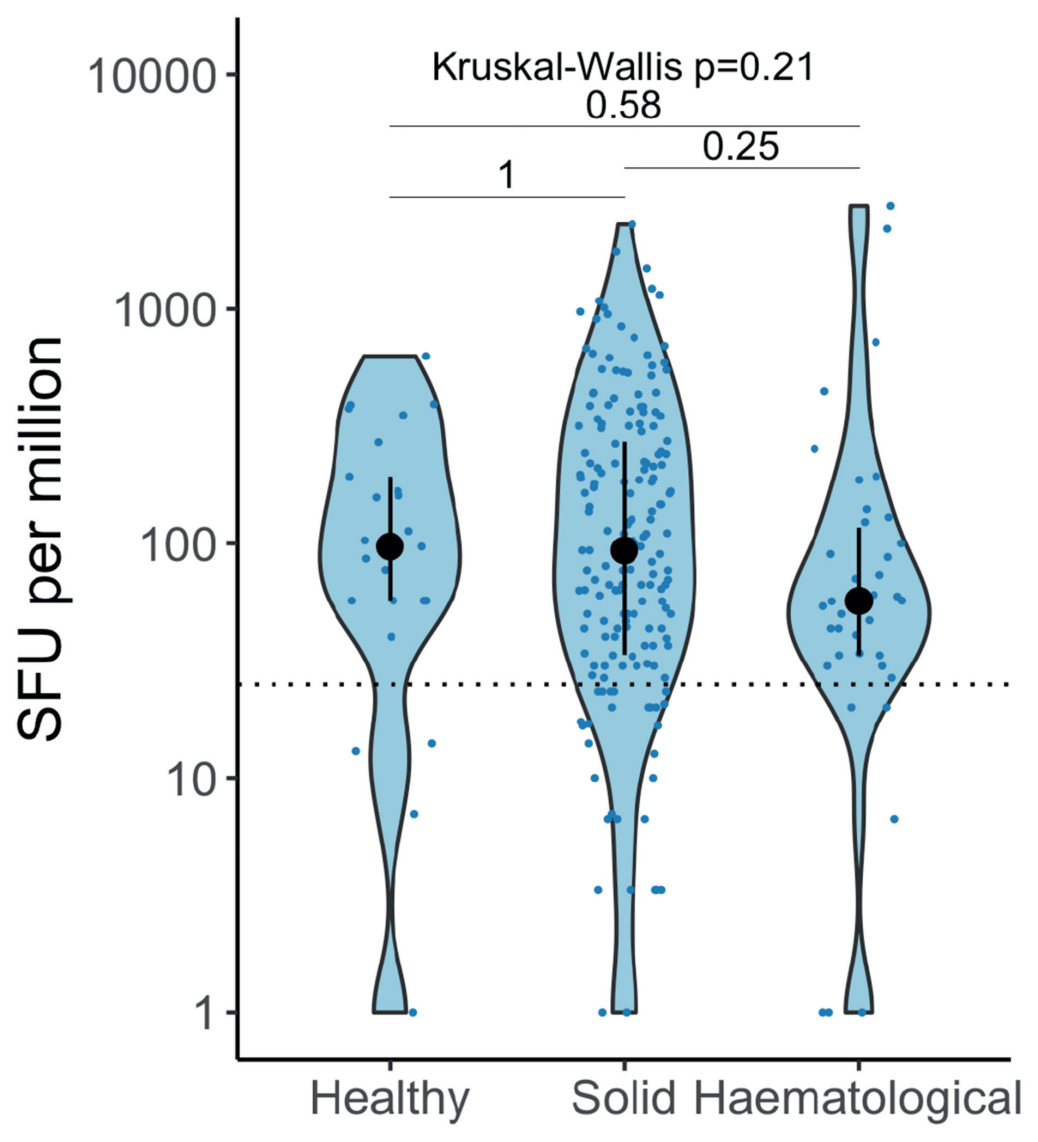


## Supplementary Material

List of Consortia Members

Supplementary Tables

## Figures and Tables

**Table 1:** Clinical and oncological characteristics of 585 vaccinated cancer patients.

Cohort characteristics, n = 585	n (%)
Age, median (IQR), years	60 (52 – 68)
Male	323 (60)
Ethnicity, white	510 (87)
**Previous SARS-CoV-2 infection and COVID-19 vaccination**	**n (%)**
Previous SARS-CoV-2*	
Any test positive	181 (31)
RT-PCR positive	82(14)
Serology positive	149 (25)
** 1^st^ COVID vaccine**	
AstraZeneca	430 (74)
Pfizer	153 (26)
Unknown	2 (0)
**Time to 2^nd^ vaccine, median (IQR), days 2^nd^ COVID vaccine**	77 (72- 78)
AstraZeneca	402 (69)
Pfizer	142 (24)
Unknown	2 (0)
**Reason for no 2^nd^ vaccine**
Death	16 (3)
Withdrew/Lost to follow-up	10 (2)
Clinical advice	7 (1)
Patient choice	6 (1)
**Oncological history**	**n (%)**
**Cancer type Solid, n = 447**
Stage I-II	55 (12)
Stage III	85 (19)
Stage IV	306 (68)
NA	1 (0)
** Haematological**	138 (24)
Concomitant medications**, within 48 hours of vaccination	
Corticosteroids, > 10mg prednisolone equivalent	29 (5)
GCSF	12 (3)
Other immunosuppression	14 (2)
Cyclosporin	6 (1)
Mycophenolate Mofetil	6 (1)
Methotrexate	1 (0)
Tacrolimus	1 (0)
**solid cancers, n = 447**	**n (%)**
** Diagnosis**
Genitourinary	93 (21)
Skin	91 (20)
Gastrointestinal	87 (19)
Thoracic	63 (14)
Breast	52 (12)
Gynaecological	27 (6)
Head and Neck	13 (3)
Other	21 (5)
**Disease status (with respect to last intervention) SACT, palliative**	
CR	32 (7)
PR	80 (18)
SD	116 (26)
PD	86 (19)
Unknown	1 (0)
**SACT, neoadjuvant or radical CRT**	
CR/PR/SD	24 (5)
PD	1 (0)
Unknown	1 (0)
** Surgery**	
NED, Adjuvant SACT	74 (17)
NED, surgery alone	17 (4)
**Untreated/active surveillance**	15 (3)
Recent anti-cancer treatment*** Systemic therapy	
Chemotherapy, <28 days	104 (23)
Targeted therapy, <28 days	145 (32)
Anti-PD(L)1 ± anti-CTLA4, <183 days	109 (24)
Endocrine therapy, <28 days	20 (4)
No SACT <28 days; no CPI <112 days	145 (32)
**Local therapy**	
Surgery, <28 days	12 (3)
Radiotherapy, <28 days	20 (4)
**Active IRAEs,** secondary to CPI	38 (9)
**Haematological malignancies, n= 138**	**n (%)**
**Diagnosis**	
Lymphoma	53 (38)
Myeloma	36 (26)
Acute leukaemia	25 (18)
CLL	16 (12)
MDS & MPN	7 (5)
Aplastic anaemia	1 (1)
**Disease status**	
MRD/CR	72 (52)
Partial remission	34 (25)
SD	5 (4)
PD/relapse/untreated acute presentation	27(20)
**Anti-cancer treatment**	
Chemotherapy, <28 days	19 (14)
Targeted therapy, <28 days	55 (40)
Anti-CD20 therapy, <12 months	26 (19)
CAR-T, <6 months	3 (2)
No SACT <28 days; no SCT or anti-CD20 <6 months	64 (46)
**Haematologic stem cell transplant**	
Any previous stem cell transplant	58 (39)
Time from transplant, median (IQR), days	855 (215-1602)
Allograft, <6 months	7 (5)
Autograft, <6 months	2 (1)
GVHD ongoing at 1^st^ vaccination	18 (13)
**Non-oncological medical history**	**n (%)**
**Past medical history**	
no PMHx	188 (32)
Obesity, BMI >30	130 (22)
HTN	121 (21)
Diabetes Melitus	54 (9)
Inflammatory/Autoimmune	38 (6)
PVD/IHD/CVD	32 (5)
Previous history cancer	63 (11)

* As some patients did not seroconvert following prior infection, our laboratory definition of previous SARS-CoV- 2 was determined by either prior PCR and/or standard of care or laboratory anti-S1 IgG ELISA (see methods) and some patients have >1 testing modality positive.

** Significant corticosteroid exposure was >10mg prednisolone for at least 7 days duration and given within 48 hours of vaccination. Significant GCSF exposure was within 48 hours of vaccination or 5-days if pegylated preparation was used.

*** SACT was considered within 28 days of last administration with the exception of CPI where treatment within 183 days was considered given prolonged receptor occupancy following administration^42^

BMI, body mass index; CAR-T, chimeric antigen receptor T-cell; CLL, chronic lymphocytic leukaemia; CPI, checkpoint inhibitor; CR, complete response; CRT, chemoradiation; CVD, cerebrovascular disease; GCSF, granulocyte-colony stimulating factor; GVHD, graft versus host disease; HTN, hypertension; IRAE, immune related adverse event secondary to CPI therapy; IHD, ischaemic heart disease; IQR, interquartile range; MDS, myelodysplastic syndrome; MPN, myeloproliferative neoplasm; MRD, minimal residual disease; NA, not available; NED, no evidence of disease; PD, progressive disease; PMHx, past medical history; PR, partial response; PVD, peripheral vascular disease; RT-PCR, reverse transcription polymerase chain reaction; SACT, systemic anticancer therapy; SCT, stem cell transplant; SD, stable disease.

## Data Availability

All requests for raw and analysed data, and CAPTURE study protocol will be reviewed by the CAPTURE Trial Management Team, Skin and Renal Clinical Trials Unit, The Royal Marsden NHS Foundation Trust (CAPTURE@rmh.nhs.uk) to determine if the request is subject to confidentiality and data protection obligations. Materials used in this study will be made available upon request. There are restrictions to the availability based on limited quantities. Response to any request for data and/or materials will be given within a 28 day period. Data and materials that can be shared would then be released upon completion of a material transfer agreement.

## Reporting Summary

Further information on research design is available in the Nature Research Reporting Summary linked to this article.

## Code availability

No unpublished code was used in this study.
