# Supplementary material for: Adaptive immunity and neutralizing antibodies against SARS-CoV-2 variants of concern following vaccination in patients with cancer: The CAPTURE study
Source: Nat Cancer. Author manuscript; Available in PMC 2021 Dec 22. (PMC7612125; doi:10.1038/s43018-021-00274-w)
Supplement: Supplementary Tables [file EMS140022-supplement-Supplementary_Tables.pdf]

Supplementary Table 1: comparison of patients recruited compared to those lost to follow-up or not receiving 2nd dose

	CAPTURE recruited, n = 585	No follow-up, n = 41	p- value	CAPTURE, received 2nd dose n= 546	CAPTURE, no 2nd dose, n=39	p-value[b]
Age, median (range)	60 (52-58)	67 (52-70)	0.196	60 (52-68)	58 (51-67)	0.217
Sex[a]	323 (55)	20 (48)	0.523	300 (55)	23 (59)	0.747
Ethnicity	510 (87)	31 (76)	0.122	480 (88)	30 (77)	0.399
Previous COVID	181 (30)	13 (31)	0.84	170 (31)	11 (28)	0.425
1st vaccine given						
PZ	153 (26)	12 (29)		144 (26)	9 (23)	
AZ	430 (74)	29 (71)	0.85	400 (73)	30 (77)	0.835
Unknown	2 (0)	0 (0)		2 (0)	0 (0)	
Cancer diagnosis						
Solid	447 (76)	37 (90)	0.06	422 (77)	25 (64)	0.09
Haematological	138 (24)	4 (10)		124 (23)	14 (36)	

[a]Values are n (%) unless otherwise stated.
[b]Comparison was made by chi-squared test or Mann-Whitney-U test as appropriate. P-value <0.05 was considered significant.

Supplementary Table 2: Number and percentages of patients, samples and assays at each sampling timepoint

	**Solid**		**Haematological**	
	Previous infection	Infection naïve	Previous infection	Infection naïve
**Patients (n)**	139	308	42	96
**Baseline (n)**	126	273	25	58
ELISA	122 (97)	270 (99)	25 (100)	58 (100)
Neutralising	110 (87)	262 (96)	23 (92)	56 (97)
ELISPOT	62 (49)	136 (50)	8 (32)	29 (50)
**FU1 (n)**	131	236	36	89
ELISA	128 (98)	234 (99)	36 (100)	89 (100)
Neutralising	128 (98)	232 (98)	35 (97)	84 (94)
ELISPOT	76 (58)	161 (68)	12 (33)	34 (38)
**FU2 (n)**	97	192	24	64
ELISA	96 (99)	192 (100)	24 (100)	64 (100)
Neutralising	92 (95)	189 (98)	23 (96)	64 (100)
ELISPOT	42 (43)	98 (51)	7 (29)	24 (38)
**FU3 (n)**	116	235	32	79
ELISA	115 (99)	234 (99)	32 (100)	78 (99)
Neutralising	114 (98)	232 (99)	30 (94)	75 (96)
ELISPOT	58 (50)	130 (55)	11 (34)	30 (38)

Numbers are n (%), FU1, 21-56 days post first-vaccine; FU2, 14-28 days prior to second-vaccine; FU3, 14-28 days post second-vaccine

Supplementary Table 3: Baseline comparison of clinical characteristics in 585 vaccinated patients.

	**Cancer Diagnosis**			**Vaccine Type**			**ELISPOT Assay**		
	Solid n= 447	Haematological n= 138	p-value	AZ n= 430	PZ n= 153	p-value	Yes n= 337	No n= 248	p-value
Age, median (range)	60 (19-87)	60 (18-85)	0.1986	59 (18-87)	63 (19-85)	**<0.001**	62 (19-85)	59 (18- 87)	0.06
Sex, male	240 (55)	83 (60)	**0.01**	239 (56)	83 (54)	0.949	189 (56)	148 (60)	0.682
Ethnicity, white	399 (89)	111 (80)	0.799	375 (87)	133 (87)	0.910	301 (89)	209 (84)	0.09
Previous SARS-CoV-2[b], yes	139 (31)	42 (30)	0.376	116 (27)	65 (42)	**0.001**	90 (27)	91 (37)	**0.01**
**1st vaccine given**									
Pfizer	105 (23)	48 (35)		-	-	-			
AstraZeneca	340 (76)	90 (65)	**0.02**	**-**	**-**	**-**			
Unknown	2 (0)	0 (0)		-	-	-			
ELISPOT, yes	279 (62)	58 (42)	**<0.001**						

Values are n (%) unless otherwise stated. Significance testing was with Chi-squared test, P-value <0.05 was considered significant.

[a] Previous COVID-19 determined by our laboratory definition of previous positive PCR or lateral flow test and/or detectable S1-reactive IgG antibodies prior to 1st vaccination

AZ, Oxford/AstraZeneca vaccine; PZ, Pfizer vaccine

Supplementary Table 4: Concordance of seroconversion and neutralising response in infection naïve patients

			Neutralising response											
			**WT**			**Alpha**			**Beta**			**Delta**		
			Absent	Detected	P-value	Absent	Detected	P-value	Absent	Detected	P-value	Absent	Detected	P-value
**S1-reactive antibodies**	**Post-first dose**	Absent	88.6 (140)	44.4 (68)	<0.001	76.4 (204)	9.1 (4)	<0.001	71.7 (203)	17.9 (5)	<0.001	72.9 (188)	16.7 (4)	<0.001
		Detected	11.4 (18)	55.6 (85)		23.6 (63)	90.9 (40)		28.3 (80)	82.1 (23)		27.1 (70)	83.3 (20)	
	**Post-2nd dose**	Absent	82.4 (42)	12.0 (30)	<0.001	50.9 (59)	7.0 (13)	<0.001	43.0 (61)	6.9 (11)	<0.001	44.9 (62)	6.1 (10)	<0.001
		Present	17.6 (9)	88.0 (220)		49.1 (57)	93.0 (172)		57.0 (81)	93.1 (148)		55.1 (76)	93.9 (153)	

All data are percentages of patients with either present or absent antibody response at each corresponding timepoint and number of patients in brackets, NAb with IC50 titres <40 was considered absent. Significance was tested by Chi-Square test. WT, wild type

Supplementary Table 5: Ordinal logistic regression models for all cancer patients

**Model 1**	**NAbT post first dose ~ Malignancy type + Vaccine type + Previous infection + Age + Sex**																
		**WT**				**Alpha**				**Beta**				**Delta**			
	**Factor**	**Coef**	**SE**	**WALD Z**	**P value**	**Coef**	**SE**	**WALD Z**	**P value**	**Coef**	**SE**	**WALD Z**	**P value**	**Coef**	**SE**	**WALD Z**	**P value**
	Malignancy type (Solid)	1.24	0.23	5.40	<0.0001	1.20	0.31	3.92	<0.0001	1.18	0.32	3.69	0.0002	1.53	0.36	4.20	<0.0001
	No. of comorbidities	-0.14	0.11	-1.29	0.20	-0.13	0.14	-0.93	0.35	-0.04	0.15	-0.24	0.81	-0.19	0.17	-1.11	0.27
	Vaccine type (Pfizer)	-0.55	0.22	-2.46	0.01	-0.29	0.28	-1.06	0.29	-0.15	0.29	-0.51	0.61	-0.05	0.31	-0.15	0.88
	Previous Infection	2.35	0.22	10.81	<0.0001	2.75	0.24	11.56	<0.0001	2.90	0.26	11.29	<0.0001	2.91	0.27	10.63	<0.0001
	Age	-0.01	0.01	-0.81	0.42	-0.01	0.01	-1.19	0.24	-0.004	0.01	-0.42	0.68	-0.02	0.01	-1.71	0.09
	Sex (Male)	-0.32	0.18	-1.75	0.08	-0.04	0.23	-0.17	0.87	0.17	0.24	0.71	0.48	-0.10	0.26	-0.39	0.70
	**ANOVA**	**WT**			**Alpha**				**Beta**					**Delta**			
	**Factor**	**Chi-Square**	**d.f.**	**P**		**Chi-Square**	**d.f.**	**P**		**Chi-Square**	**d.f.**	**P**		**Chi-Square**	**d.f.**	**P**	
	Malignancy type (Solid)	**29.17**	**1.00**	**<.0001**		**15.39**	**1.00**	**0.0001**		**13.62**	**1.00**	**0.0002**		**17.64**	**1.00**	**<.0001**	
	No. of comorbidities	1.66	1.00	0.20		0.87	1.00	0.35		0.06	1.00	0.81		1.24	1.00	0.27	
	Vaccine type (Pfizer)	**6.04**	**1.00**	**0.01**		1.13	1.00	0.29		0.26	1.00	0.61		0.02	1.00	0.88	
	Previous Infection	**116.96**	**1.00**	**<.0001**		**133.59**	**1.00**	**<.0001**		**127.57**	**1.00**	**<.0001**		**112.98**	**1.00**	**<.0001**	
	Age	0.66	1.00	0.42		1.41	1.00	0.24		0.18	1.00	0.68		**2.92**	**1.00**	**0.09**	
	Sex (Male)	3.06	1.00	0.08		0.03	1.00	0.87		0.50	1.00	0.48		0.15	1.00	0.70	

**Model 2**	**NAbT post second dose ~ Malignancy type + Vaccine type + Previous infection + Age + Sex**																
		**WT**				**Alpha**				**Beta**				**Delta**			
	**Factor**	**Coef**	**S.E.**	**WALD Z**	**P value**	**Coef**	**SE**	**WALD Z**	**P value**	**Coef**	**S.E.**	**WALD Z**	**P value**	**Coef**	**S.E.**	**WALD Z**	**P value**
	Malignancy type (Solid)	1.85	0.25	7.52	<0.0001	1.6627	0.2463	6.75	<0.0001	1.81	0.26	7.04	<0.0001	1.53	0.25	6.19	<0.0001
	No. of comorbidities	-0.16	0.11	-1.46	0.15	-0.0939	0.1079	-0.87	0.3844	-0.04	0.11	-0.41	0.68	-0.05	0.11	-0.43	0.67
	Vaccine type (Pfizer)	0.82	0.23	3.59	0.0003	1.1816	0.2248	5.26	<0.0001	1.17	0.22	5.22	<0.0001	1.17	0.22	5.34	<0.0001
	Previous Infection	1.33	0.22	6.02	<0.0001	1.698	0.2147	7.91	<0.0001	1.69	0.22	7.74	<0.0001	1.41	0.21	6.78	<0.0001
	Age	-0.02	0.01	-3.04	0.002	-0.027	0.0076	-3.56	0.0004	-0.03	0.01	-4.41	<0.0001	-0.02	0.01	-2.87	0.004
	Sex (Male)	0.12	0.19	0.64	0.52	0.0056	0.1863	0.03	0.976	-0.20	0.19	-1.06	0.29	-0.08	0.19	-0.42	0.68
	**ANOVA**	**WT**				**Alpha**				**Beta**				**Delta**			
	**Factor**	**Chi-Square**	**d.f.**	**P value**		**Chi-Square**	**d.f.**	**P value**		**Chi-Square**	**d.f.**	**P value**		**Chi-Square**	**d.f.**	**P value**	
	Malignancy type (Solid)	**56.59**	**1.00**	**<.0001**		**45.57**	**1.00**	**<.0001**		**49.61**	**1.00**	**<.0001**		**38.35**	**1.00**	**<.0001**	
	No. of comorbidities	2.12	1.00	0.15		0.76	1.00	0.38		0.17	1.00	0.68		0.18	1.00	0.67	
	Vaccine type (Pfizer)	**12.89**	**1.00**	**0.0003**		**27.63**	**1.00**	**<.0001**		**27.29**	**1.00**	**<.0001**		**28.56**	**1.00**	**<.0001**	
	Previous Infection	**36.26**	**1.00**	**<.0001**		**62.56**	**1.00**	**<.0001**		**59.84**	**1.00**	**<.0001**		**45.99**	**1.00**	**<.0001**	
	Age	**9.25**	**1.00**	**0.002**		**12.64**	**1.00**	**0.0004**		**19.44**	**1.00**	**<.0001**		**8.22**	**1.00**	**0.004**	
	Sex (Male)	0.41	1.00	0.52		0.00	1.00	0.98		1.12	1.00	0.29		0.17	1.00	0.68	

NAbT were binned into three categories: Undetectable/low (IC50 titres <40), medium (40-256), high (>256)

NAbT, Neutralising antibody titers

Supplementary Table 6: Ordinal logistic regression models for patients with haematological malignancy

Model 1 NAbT post first dose ~ Diagnosis + Chemotherapy + Targeted therapy + anti-CD20 + Stem cell transplant + CAR-T therapy + Previous_infection + Vaccine type + Age

	WT				Alpha				Beta				Delta			
**Factor**	**Coef**	**S.E.**	**WALD Z**	**P value**	**Coef**	**S.E.**	**WALD Z**	**P value**	**Coef**	**S.E.**	**WALD Z**	**P value**	**Coef**	**S.E.**	**WALD Z**	**P value**
Diagnosis (CLL)	-0.71	0.91	-0.78	0.44	-1.42	1.39	-1.02	0.31	0.33	1.23	0.27	0.79	-1.76	1.59	-1.10	0.27
Diagnosis (MDS/MPN)	-9.78	39.60	-0.25	0.81	-9.14	47.15	-0.19	0.85	-8.67	48.94	-0.18	0.86	-8.92	52.23	-0.17	0.86
Diagnosis (Lymphoma)	-1.18	0.65	-1.82	0.07	-0.37	0.78	-0.47	0.64	-0.58	0.96	-0.61	0.54	-0.18	1.05	-0.17	0.86
Diagnosis (Myeloma)	-0.27	0.64	-0.43	0.67	-1.60	0.90	-1.79	0.07	-1.14	0.92	-1.24	0.22	-1.87	1.22	-1.53	0.13
Chemotherapy (28 days)	0.02	0.80	0.03	0.98	-0.32	0.97	-0.33	0.74	0.93	0.88	1.05	0.29	-0.24	1.21	-0.20	0.85
Targeted therapy (28 days)	-0.39	0.50	-0.77	0.44	-0.04	0.67	-0.05	0.96	-0.59	0.72	-0.81	0.42	0.55	0.95	0.58	0.57
anti-CD20 therapy (12 month	-3.40	1.24	-2.74	0.01	-2.41	1.23	-1.97	0.05	-1.93	1.30	-1.49	0.14	-1.81	1.32	-1.37	0.17
Stem cell tranplant (6 month	0.73	1.11	0.66	0.51	0.84	1.14	0.74	0.46	0.71	1.16	0.61	0.54	0.24	1.46	0.17	0.87
Car-T therapy	-8.51	50.28	-0.17	0.87	-8.84	57.30	-0.15	0.88	-8.30	62.17	-0.13	0.89	-8.95	58.86	-0.15	0.88
Previous Infection	2.51	0.53	4.77	<0.0001	2.94	0.66	4.46	<0.0001	2.73	0.69	3.94	<0.0001	4.24	1.16	3.66	0.0003
Vaccine type (Pfizer)	-1.21	0.55	-2.22	0.03	-0.20	0.68	-0.29	0.77	-0.07	0.68	-0.11	0.92	0.63	0.89	0.71	0.48
Age	0.00	0.02	0.21	0.83	0.00	0.02	0.14	0.89	0.02	0.02	0.99	0.32	0.00	0.03	-0.12	0.90
**ANOVA**	**WT**				**Alpha**				**Beta**				**Delta**			
**Factor**	**Chi-Square**	**d.f.**	**P value**		**Chi-Square**	**d.f.**	**P value**		**Chi-Square**	**d.f.**	**P value**		**Chi-Square**	**d.f.**	**P value**	
Diagnosis	3.81	4.00	0.43		3.56	4.00	0.47		2.67	4.00	0.61		3.03	4.00	0.55	
Chemotherapy (28 days)	0.00	1.00	0.98		0.11	1.00	0.74		1.11	1.00	0.29		0.04	1.00	0.85	
Targeted therapy (28 days)	0.59	1.00	0.44		0.00	1.00	0.96		0.66	1.00	0.42		0.33	1.00	0.57	
anti-CD20 therapy (12 month	**7.53**	**1.00**	**0.01**		**3.86**	**1.00**	**0.05**		2.21	1.00	0.14		1.88	1.00	0.17	
Stem cell tranplant (6 month	0.43	1.00	0.51		0.54	1.00	0.46		0.37	1.00	0.54		0.03	1.00	0.87	
Car-T therapy	0.03	1.00	0.87		0.02	1.00	0.88		0.02	1.00	0.89		0.02	1.00	0.88	
Previous Infection	**22.74**	**1.00**	**<.0001**		**19.90**	**1.00**	**<0.0001**		**15.56**	**1.00**	**0.0001**		**13.36**	**1.00**	**0.0003**	
Vaccine type (Pfizer)	**4.91**	**1.00**	**0.03**		0.09	1.00	0.77		0.01	1.00	0.92		0.50	1.00	0.48	
Age	0.05	1.00	0.83		0.02	1.00	0.89		0.98	1.00	0.32		0.01	1.00	0.90	

Model 2 NAbT post second dose ~ Diagnosis + Chemotherapy + Targeted therapy + anti-CD20 + Stem cell transplant + CAR-T therapy + Previous infection + Vaccine type + Age

	WT				Alpha				Beta				Delta			
**Factor**	**Coef**	**S.E.**	**WALD Z**	**P value**	**Coef**	**S.E.**	**WALD Z**	**P value**	**Coef**	**S.E.**	**WALD Z**	**P value**	**Coef**	**S.E.**	**WALD Z**	**P value**
Diagnosis (CLL)	-1.31	0.91	-1.44	0.15	-2.38	1.29	-1.84	0.07	-1.80	1.16	-1.54	0.12	-1.10	0.96	-1.15	0.25
Diagnosis (MDS/MPN)	-1.25	0.95	-1.31	0.19	-0.32	0.94	-0.34	0.73	-0.74	0.98	-0.76	0.45	-0.50	0.94	-0.53	0.60
Diagnosis (Lymphoma)	-0.61	0.64	-0.95	0.34	-0.47	0.64	-0.74	0.46	-0.38	0.66	-0.58	0.56	-0.82	0.65	-1.27	0.21
Diagnosis (Myeloma)	1.25	0.73	1.70	0.09	0.54	0.72	0.75	0.45	-0.15	0.78	-0.19	0.85	-0.22	0.72	-0.31	0.76
Chemotherapy (28 days)	-0.82	0.64	-1.28	0.20	-0.84	0.66	-1.27	0.20	-1.54	0.88	-1.76	0.08	-0.91	0.76	-1.21	0.23
Targeted therapy (28 days)	-0.25	0.53	-0.48	0.63	0.19	0.57	0.33	0.74	-0.04	0.62	-0.06	0.95	-0.20	0.56	-0.35	0.73
anti-CD20 therapy (12 month	-1.94	0.89	-2.17	0.03	-1.91	1.15	-1.66	0.10	-2.52	1.21	-2.08	0.04	-1.68	1.14	-1.47	0.14
SCT (6 months)	-7.36	35.80	-0.21	0.84	-6.43	33.33	-0.19	0.85	-7.23	57.51	-0.13	0.90	-6.34	35.38	-0.18	0.86
Car-T	-1.08	1.39	-0.77	0.44	-0.48	1.31	-0.37	0.71	-8.87	40.39	-0.22	0.83	-0.22	1.32	-0.17	0.86
Previous Infection	1.18	0.46	2.59	0.01	1.24	0.45	2.72	0.01	1.41	0.50	2.81	0.005	0.91	0.45	2.01	0.04
Vaccine type (Pfizer)	-0.02	0.46	-0.04	0.97	-0.02	0.49	-0.03	0.97	0.82	0.53	1.53	0.13	0.23	0.50	0.46	0.65
Age	-0.04	0.01	-2.93	0.003	-0.04	0.01	-2.63	0.01	-0.04	0.01	-2.88	0.004	-0.03	0.01	-2.41	0.02
**ANOVA**	**WT**				**Alpha**				**Beta**				**Delta**			
**Factor**	**Chi-Square**	**d.f.**	**P value**		**Chi-Square**	**d.f.**	**P value**		**Chi-Square**	**d.f.**	**P value**		**Chi-Square**	**d.f.**	**P value**	
Diagnosis	**15.61**	**4**	**0.0036**		7.72	4	0.1025		3.47	4	0.4822		2.77	4	0.5971	
Chemotherapy (28 days)	1.64	1	0.1998		1.62	1	0.2037		3.09	1	0.0787		1.46	1	0.2272	
Targeted therapy (28 days)	0.23	1	0.6335		0.11	1	0.7382		0	1	0.9542		0.12	1	0.727	
anti-CD20 therapy (12 month	**4.69**	**1**	**0.0303**		2.75	1	0.097		**4.33**	**1**	**0.0375**		2.16	1	0.1417	
SCT (6 months)	0.04	1	0.8372		0.04	1	0.847		0.02	1	0.9		0.03	1	0.8578	
Car-T	0.6	1	0.4393		0.14	1	0.7125		0.05	1	0.8262		0.03	1	0.8648	
Previous Infection	**6.7**	**1**	**0.0096**		**7.39**	**1**	**0.0066**		**7.9**	**1**	**0.0049**		**4.04**	**1**	**0.0445**	
Vaccine type (Pfizer)	0.000	1	0.9654		0.0000	1	0.9741		2.33	1	0.1266		0.21	1	0.6476	
Age	**8.56**	**1**	**0.0034**		**6.93**	**1**	**0.0085**		**8.32**	**1**	**0.0039**		**5.81**	**1**	**0.0159**	

Model 3 NAbT post first dose ~ GVHD + GCSF + Steroids + Age + Vaccine type + Previous_infection

	WT				Alpha				Beta				Delta			
**Factor**	**Coef**	**S.E.**	**WALD Z**	**P value**	**Coef**	**S.E.**	**WALD Z**	**P value**	**Coef**	**S.E.**	**WALD Z**	**P value**	**Coef**	**S.E.**	**WALD Z**	**P value**
GVHD	1.25	0.57	2.17	0.03	0.23	0.74	0.32	0.75	0.57	0.82	0.70	0.49	0.98	0.94	1.04	0.30
GCSF	0.70	0.91	0.76	0.45	0.11	1.31	0.09	0.93	2.04	1.16	1.76	0.08	1.06	1.43	0.74	0.46
Steroids	-0.96	0.87	-1.10	0.27	-0.66	1.27	-0.52	0.61	-8.21	37.73	-0.22	0.83	-8.60	39.38	-0.22	0.83
Age	0.01	0.01	0.78	0.43	-0.01	0.02	-0.32	0.75	0.01	0.02	0.60	0.55	-0.01	0.02	-0.60	0.55
Vaccine type (Pfizer)	-1.33	0.49	-2.69	0.01	-0.47	0.61	-0.77	0.44	-0.24	0.65	-0.37	0.71	0.42	0.79	0.53	0.59
Previous Infection	2.13	0.44	4.86	<0.0001	2.48	0.56	4.45	<0.0001	2.69	0.64	4.18	<0.0001	3.87	1.09	3.54	0.0004
**ANOVA**	**WT**				**Alpha**				**Beta**				**Delta**			
**Factor**	**Chi-Square**	**d.f.**	**P value**		**Chi-Square**	**d.f.**	**P value**		**Chi-Square**	**d.f.**	**P value**		**Chi-Square**	**d.f.**	**P value**	
GVHD	**4.70**	**1.00**	**0.03**		0.10	1.00	0.75		0.49	1.00	0.49		1.08	1.00	0.30	
GCSF	0.58	1.00	0.45		0.01	1.00	0.93		3.08	1.00	0.08		0.55	1.00	0.46	
Steroids	1.21	1.00	0.27		0.27	1.00	0.61		0.05	1.00	0.83		0.05	1.00	0.83	
Age	0.61	1.00	0.43		0.10	1.00	0.75		0.36	1.00	0.55		0.35	1.00	0.55	
Vaccine type (Pfizer)	**7.24**	**1.00**	**0.01**		0.60	1.00	0.44		0.13	1.00	0.71		0.28	1.00	0.59	
Previous Infection	**23.62**	**1.00**	**<.0001**		**19.79**	**1.00**	**<.0001**		**17.46**	**1.00**	**<.0001**		**12.55**	**1.00**	**0.0004**	

Model 4 NAbT post second dose ~ GVHD + GCSF + Steroids + Age + Vaccine type + Previous_infection

	WT				Alpha				Beta				Delta			
**Factor**	**Coef**	**S.E.**	**WALD Z**	**P value**	**Coef**	**S.E.**	**WALD Z**	**P value**	**Coef**	**S.E.**	**WALD Z**	**P value**	**Coef**	**S.E.**	**WALD Z**	**P value**
GVHD	-0.04	0.55	-0.07	0.95	0.05	0.55	0.09	0.93	-0.13	0.61	-0.21	0.83	0.39	0.56	0.70	0.48
GCSF	0.17	1.04	0.17	0.87	-0.09	1.22	-0.08	0.94	0.32	1.27	0.25	0.80	0.01	1.24	0.01	0.99
Steroids	-0.13	1.00	-0.13	0.90	-0.13	0.95	-0.13	0.89	-0.84	1.01	-0.83	0.41	0.16	0.94	0.17	0.86
Age	-0.03	0.01	-2.06	0.04	-0.03	0.01	-2.37	0.02	-0.04	0.01	-3.09	0.00	-0.03	0.01	-2.36	0.02
Vaccine type (Pfizer)	-0.53	0.41	-1.31	0.19	-0.49	0.44	-1.11	0.27	0.19	0.46	0.41	0.68	-0.24	0.45	-0.53	0.60
Previous Infection	1.27	0.43	2.95	0.003	1.36	0.43	3.14	0.002	1.58	0.47	3.40	0.001	1.05	0.44	2.37	0.02
**ANOVA**	**WT**				**Alpha**				**Beta**				**Delta**			
**Factor**	**Chi-Square**	**d.f.**	**P value**		**Chi-Square**	**d.f.**	**P value**		**Chi-Square**	**d.f.**	**P value**		**Chi-Square**	**d.f.**	**P value**	
GVHD	0	1	0.945		0.01	1	0.9322		0.04	1	0.8345		0.49	1	0.482	
GCSF	0.03	1	0.8685		0.01	1	0.9385		0.06	1	0.8015		0	1	0.9921	
Steroids	0.02	1	0.8997		0.02	1	0.8933		0.69	1	0.4074		0.03	1	0.8625	
Age	**4.25**	**1**	**0.0393**		**5.59**	**1**	**0.018**		**9.54**	**1**	**0.002**		**5.56**	**1**	**0.0183**	
Vaccine type (Pfizer)	1.71	1	0.191		1.22	1	0.2686		0.17	1	0.6796		0.28	1	0.5963	
Previous Infection	**8.68**	**1**	**0.0032**		**9.85**	**1**	**0.0017**		**11.53**	**1**	**0.0007**		**5.62**	**1**	**0.0178**	

NAbT were binned into three categories: Undetectable/low (IC50 titres <40), medium (40-256), high (>256)

NAbT, Neutralising antibody titers

CLL, chronic lymphocytic leukaemia

MDS, myelodysplastic syndrome; MPN, myeloproliferative neoplasm

CAR-T, chimeric antigen receptor T-cell

GVHD, Graft versus host disease

GCSF, granulocyte colony-stimulating factor

Supplementary Table 7: Ordinal logistic regression models for patients with solid malignancies

Model 1 **NAbT post first dose ~ Diagnosis + Cancer Status + Cancer Stage + Chemotherapy + targeted therapy + Endocrine therapy + Checkpoint inhibitors + Radiotherapy + Previous_infection + Vaccine type + Age**

	WT				Alpha				Beta				Delta			
**Factor**	**Coef**	**S.E.**	**WALD Z**	**P value**	**Coef**	**S.E.**	**WALD Z**	**P value**	**Coef**	**S.E.**	**WALD Z**	**P value**	**Coef**	**S.E.**	**WALD Z**	**P value**
Diagnosis (Gastrointestinal)	-0.36	0.45	-0.80	0.42	-0.68	0.52	-1.32	0.19	-0.18	0.51	-0.35	0.73	-0.65	0.56	-1.16	0.25
Diagnosis (Genitourinary)	-0.24	0.45	-0.54	0.59	-0.79	0.54	-1.46	0.14	-0.55	0.55	-1.01	0.31	-1.16	0.58	-2.00	0.05
Diagnosis (Gynaecological)	0.48	0.58	0.83	0.40	-0.19	0.68	-0.28	0.78	-0.10	0.71	-0.14	0.89	-0.18	0.71	-0.26	0.79
Diagnosis (Head and Neck)	0.18	0.72	0.25	0.80	0.80	0.78	1.02	0.31	0.29	0.86	0.34	0.73	-0.56	0.90	-0.62	0.53
Diagnosis (Skin)	-0.07	0.49	-0.14	0.89	-0.45	0.57	-0.77	0.44	-0.42	0.60	-0.70	0.49	-1.07	0.63	-1.70	0.09
Diagnosis (Solid other)	0.56	0.69	0.80	0.42	0.25	0.82	0.31	0.76	-0.08	0.91	-0.08	0.93	-0.44	0.97	-0.45	0.65
Diagnosis (Thoracic)	0.24	0.48	0.50	0.62	-0.69	0.58	-1.19	0.23	-0.62	0.60	-1.03	0.30	-1.02	0.67	-1.51	0.13
Status (CR)	0.89	0.78	1.14	0.25	0.94	1.00	0.94	0.35	0.69	1.10	0.62	0.53	1.62	1.33	1.22	0.22
Status (NACRTx)	-0.85	0.83	-1.03	0.30	-0.87	1.09	-0.80	0.43	-1.14	1.19	-0.96	0.33	0.90	1.39	0.65	0.52
Status (NED)	0.39	0.73	0.54	0.59	0.57	0.90	0.63	0.53	0.52	1.01	0.51	0.61	2.26	1.26	1.79	0.07
Status (PD)	0.52	0.69	0.75	0.45	0.91	0.87	1.05	0.30	1.02	0.97	1.05	0.29	2.07	1.21	1.71	0.09
Status (PR)	0.31	0.71	0.43	0.66	0.25	0.90	0.28	0.78	0.34	1.00	0.34	0.73	1.50	1.22	1.23	0.22
Status (SD)	0.50	0.69	0.73	0.47	0.71	0.88	0.80	0.42	0.91	0.98	0.93	0.35	1.55	1.21	1.28	0.20
Stage (III)	1.01	0.44	2.30	0.02	0.55	0.52	1.06	0.29	0.10	0.55	0.19	0.85	1.13	0.60	1.89	0.06
Stage IV)	0.27	0.49	0.54	0.59	-0.30	0.58	-0.52	0.60	-0.41	0.63	-0.65	0.52	0.65	0.71	0.91	0.36
Chemotherapy (28 days)	0.00	0.30	0.02	0.99	0.04	0.35	0.13	0.90	0.03	0.36	0.09	0.93	-0.14	0.38	-0.37	0.71
Targeted Therapy (28 days)	0.28	0.25	1.13	0.26	0.51	0.30	1.71	0.09	0.01	0.32	0.03	0.97	-0.18	0.34	-0.53	0.60
Endocrine Therapy (28 days)	0.20	0.59	0.34	0.74	-0.01	0.67	-0.02	0.99	-0.22	0.69	-0.32	0.75	-0.21	0.73	-0.29	0.77
Checkpoint inhibitors (6 months)	-0.24	0.27	-0.90	0.37	-0.30	0.35	-0.86	0.39	-0.15	0.36	-0.40	0.69	0.20	0.39	0.50	0.62
Radiotherapy (28 days)	-0.23	0.55	-0.41	0.68	-1.06	0.74	-1.42	0.15	-0.81	0.81	-1.00	0.32	-0.67	0.81	-0.83	0.41
Previous Infection	2.63	0.27	9.62	<0.0001	3.08	0.30	10.26	<0.0001	3.03	0.30	9.99	<0.0001	2.98	0.32	9.20	<0.0001
Vaccine type (Pfizer)	-0.32	0.28	-1.14	0.25	-0.29	0.35	-0.83	0.41	-0.06	0.35	-0.17	0.87	-0.36	0.37	-0.96	0.34
Age	-0.01	0.01	-1.41	0.16	-0.01	0.01	-0.80	0.42	-0.01	0.01	-0.73	0.47	-0.02	0.01	-1.63	0.10

**ANOVA**	WT			Alpha			Beta			Delta		
**Factor**	**Chi-Square**	**d.f.**	**P value**	**Chi-Square**	**d.f.**	**P value**	**Chi-Square**	**d.f.**	**P value**	**Chi-Square**	**d.f.**	**P value**
Diagnosis	5.71	7	0.5744	8.63	7	0.2804	2.96	7	0.889	6.19	7	0.5179
Status	7.47	6	0.28	7.42	6	0.2839	9.35	6	0.1551	7.98	6	0.2394
Stage	**6.51**	**2**	**0.0385**	3.7	2	0.157	1.04	2	0.5934	3.71	2	0.1562
Chemotherapy (28 days)	0	1	0.9873	0.02	1	0.8986	0.01	1	0.9305	0.13	1	0.7135
Targeted Therapy (28 days)	1.27	1	0.2595	2.92	1	0.0876	0	1	0.9723	0.28	1	0.5957
Endocrine Therapy (28 days)	0.11	1	0.7355	0	1	0.9862	0.1	1	0.7524	0.09	1	0.7701
Checkpoint inhibitors (6 months)	0.81	1	0.3683	0.73	1	0.3915	0.16	1	0.6867	0.25	1	0.6161
Radiotherapy (28 days)	0.17	1	0.6818	2.03	1	0.1547	1.01	1	0.315	0.68	1	0.4091
Previous Infection	**92.45**	**1**	**<.0001**	**105.28**	**1**	**<.0001**	**99.81**	**1**	**<.0001**	**84.57**	**1**	**<.0001**
Vaccine type (Pfizer)	1.31	1	0.2522	0.69	1	0.4056	0.03	1	0.8689	0.92	1	0.3377
Age	1.99	1	0.1586	0.64	1	0.4245	0.53	1	0.4684	2.67	1	0.1023

Model 2 **NAbT post second dose ~ Diagnosis + Cancer Status + Cancer Stage + Chemotherapy + targeted therapy + Endocrine therapy + Checkpoint inhibitors + Radiotherapy + Previous_infection + Vaccine type + Age**

	WT				Alpha				Beta				Delta			
**Factor**	**Coef**	**S.E.**	**WALD Z**	**P value**	**Coef**	**S.E.**	**WALD Z**	**P value**	**Coef**	**S.E.**	**WALD Z**	**P value**	**Coef**	**S.E.**	**WALD Z**	**P value**
Diagnosis (Gastrointestinal)	-0.16	0.51	-0.31	0.75	-0.21	0.48	-0.44	0.66	-0.70	0.48	-1.47	0.14	0.03	0.46	0.07	0.94
Diagnosis (Genitourinary)	-0.64	0.51	-1.25	0.21	-1.33	0.50	-2.68	0.01	-1.30	0.50	-2.60	0.01	-0.87	0.48	-1.81	0.07
Diagnosis (Gynaecological)	0.11	0.67	0.17	0.86	-0.10	0.62	-0.16	0.87	-0.58	0.61	-0.95	0.34	0.27	0.60	0.45	0.65
Diagnosis (Head and Neck)	-0.82	0.93	-0.89	0.38	-0.21	0.85	-0.25	0.80	-0.83	0.84	-0.98	0.33	-0.08	0.79	-0.10	0.92
Diagnosis (Skin)	-0.16	0.55	-0.29	0.77	-0.45	0.52	-0.85	0.39	-0.56	0.51	-1.09	0.28	-0.13	0.50	-0.25	0.80
Diagnosis (Solid other)	0.06	0.70	0.09	0.93	0.13	0.66	0.20	0.85	0.08	0.65	0.12	0.90	0.73	0.63	1.16	0.25
Diagnosis (Thoracic)	0.14	0.58	0.25	0.81	-0.44	0.54	-0.80	0.42	-0.54	0.53	-1.01	0.31	-0.14	0.52	-0.26	0.79
Status (CR)	0.42	1.18	0.35	0.72	0.28	1.04	0.27	0.79	0.95	1.10	0.86	0.39	0.49	1.06	0.46	0.65
Status (NACRTx)	0.43	1.30	0.33	0.74	0.30	1.17	0.26	0.80	0.43	1.21	0.35	0.72	0.01	1.17	0.01	0.99
Status (NED)	0.70	1.22	0.57	0.57	0.89	1.10	0.81	0.42	1.33	1.14	1.17	0.24	0.64	1.11	0.58	0.56
Status (PD)	0.88	1.13	0.79	0.43	0.90	0.99	0.91	0.36	1.17	1.05	1.12	0.26	0.75	1.01	0.74	0.46
Status (PR)	0.89	1.15	0.78	0.44	0.55	1.01	0.55	0.58	0.62	1.06	0.58	0.56	0.38	1.03	0.37	0.71
Status (SD)	1.01	1.14	0.88	0.38	0.98	1.00	0.98	0.33	0.92	1.06	0.87	0.39	0.92	1.02	0.90	0.37
Stage (III)	0.37	0.47	0.78	0.43	0.59	0.47	1.26	0.21	0.64	0.44	1.44	0.15	0.54	0.44	1.22	0.22
Stage IV)	0.64	0.59	1.09	0.28	0.88	0.58	1.52	0.13	0.88	0.55	1.59	0.11	0.63	0.56	1.12	0.26
Chemotherapy (28 days)	0.43	0.35	1.24	0.22	0.11	0.33	0.32	0.75	0.24	0.32	0.76	0.45	0.15	0.32	0.46	0.65
Targeted Therapy (28 days)	0.17	0.28	0.61	0.54	-0.04	0.26	-0.14	0.89	0.12	0.25	0.48	0.63	0.14	0.25	0.57	0.57
Endocrine Therapy (28 days)	-0.06	0.64	-0.09	0.93	0.00	0.65	0.00	1.00	-0.58	0.65	-0.89	0.38	0.61	0.62	0.99	0.32
Checkpoint inhibitors (6 months)	0.24	0.30	0.79	0.43	-0.07	0.28	-0.23	0.82	0.23	0.28	0.82	0.41	0.03	0.28	0.10	0.92
Radiotherapy (28 days)	-0.29	1.21	-0.24	0.81	0.83	1.15	0.72	0.47	1.29	1.17	1.10	0.27	-0.50	1.14	-0.44	0.66
Previous Infection	1.49	0.28	5.30	<0.0001	2.01	0.28	7.26	<0.0001	1.88	0.27	6.94	<0.0001	1.70	0.26	6.51	<0.0001
Vaccine type (Pfizer)	1.93	0.33	5.80	<0.0001	2.25	0.32	7.09	<0.0001	1.69	0.29	5.91	<0.0001	1.96	0.29	6.74	<0.0001
Age	-0.02	0.01	-1.88	0.06	-0.02	0.01	-1.66	0.10	-0.03	0.01	-2.50	0.01	-0.01	0.01	-1.05	0.29

**ANOVA**	WT			Alpha			Beta			Delta		
**Factor**	**Chi-Square**	**d.f.**	**P value**	**Chi-Square**	**d.f.**	**P value**	**Chi-Square**	**d.f.**	**P value**	**Chi-Square**	**d.f.**	**P value**
Diagnosis	5.08	7.00	0.65	14.90	7.00	0.04	11.10	7.00	0.13	11.67	7.00	0.11
Status	2.56	6.00	0.86	5.36	6.00	0.50	6.22	6.00	0.40	5.25	6.00	0.51
Stage	1.22	2.00	0.54	2.48	2.00	0.29	2.96	2.00	0.23	1.75	2.00	0.42
Chemotherapy (28 days)	1.53	1.00	0.22	0.10	1.00	0.75	0.58	1.00	0.45	0.21	1.00	0.65
Targeted Therapy (28 days)	0.37	1.00	0.54	0.02	1.00	0.89	0.23	1.00	0.63	0.32	1.00	0.57
Endocrine Therapy (28 days)	0.01	1.00	0.93	0.00	1.00	1.00	0.79	1.00	0.38	0.98	1.00	0.32
Checkpoint inhibitors (6 months)	0.62	1.00	0.43	0.05	1.00	0.82	0.67	1.00	0.41	0.01	1.00	0.92
Radiotherapy (28 days)	0.06	1.00	0.81	0.52	1.00	0.47	1.22	1.00	0.27	0.19	1.00	0.66
Previous Infection	**28.04**	**1.00**	**<.0001**	**52.69**	**1.00**	**<.0001**	**48.21**	**1.00**	**<.0001**	**42.39**	**1.00**	**<.0001**
Vaccine type (Pfizer)	**33.69**	**1.00**	**<.0001**	**50.34**	**1.00**	**<.0001**	**34.92**	**1.00**	**<.0001**	**45.41**	**1.00**	**<.0001**
Age	3.53	1.00	0.06	2.77	1.00	0.10	**6.25**	**1.00**	**0.01**	1.11	1.00	0.29

Model 3 **NAbT post first dose ~ Other Immunosupression + GCSF + Steroids + Previous_infection + Vaccine type + Age**

	WT				Alpha				Beta				Delta			
**Factor**	**Coef**	**S.E.**	**WALD Z**	**P value**	**Coef**	**S.E.**	**WALD Z**	**P value**	**Coef**	**S.E.**	**WALD Z**	**P value**	**Coef**	**S.E.**	**WALD Z**	**P value**
Immunosupression other	0.428	1.6296	0.26	0.7928	-6.0786	38.6691	-0.16	0.8751	0.5302	1.75E+00	0.3	0.7622	-5.8582	40.5901	-0.14	0.8852
GCSF	0.388	0.816	0.48	0.6344	1.1895	0.7767	1.53	0.1257	-0.2413	0.9579	-0.25	0.8011	1.3428	0.8932	1.5	0.1328
Steroids	0.3815	0.5122	0.74	0.4563	0.184	0.5724	0.32	0.7478	-0.382	0.577	-0.66	0.5079	0.6218	0.6258	0.99	0.3204
Previous Infection	2.3589	0.2502	9.43	<0.0001	2.8183	0.2658	10.6	<0.0001	1.7543	0.2532	6.93	<0.0001	2.8673	0.2927	9.8	<0.0001
Vaccine type (Pfizer)	-0.3225	0.2586	-1.25	0.2123	-0.3018	0.3174	-0.95	0.3416	1.4974	0.2657	5.64	<0.0001	-0.158	0.3431	-0.46	0.6452
Age	-0.0152	0.0095	-1.6	0.1103	-0.017	0.0115	-1.48	0.1386	-0.0295	0.0095	-3.09	0.002	-0.0249	0.0124	-2.01	0.0442

**ANOVA**	WT			Alpha			Beta			Delta		
**Factor**	**Chi-Square**	**d.f.**	**P value**	**Chi-Square**	**d.f.**	**P value**	**Chi-Square**	**d.f.**	**P value**	**Chi-Square**	**d.f.**	**P value**
Immunosupression other	0.07	1	0.7928	0.02	1	0.8751	0.09	1	0.7622	0.02	1	0.8852
GCSF	0.23	1	0.6344	2.35	1	0.1257	0.06	1	0.8011	2.26	1	0.1328
Steroids	0.55	1	0.4563	0.1	1	0.7478	0.44	1	0.5079	0.99	1	0.3204
Previous Infection	**88.9**	**1**	**<.0001**	**112.43**	**1**	**<.0001**	**48.02**	**1**	**<.0001**	**95.99**	**1**	**<.0001**
Vaccine type (Pfizer)	1.56	1	0.2123	0.9	1	0.3416	**31.77**	**1**	**<.0001**	0.21	1	0.6452
Age	2.55	1	0.1103	2.19	1	0.1386	**9.55**	**1**	**0.002**	**4.05**	**1**	**0.0442**

Formula **NAbT post second dose ~ Other Immunosupression + GCSF + Steroids + Previous_infection + Cvaccine type + Age**

	WT				Alpha				Beta				Delta			
**Factor**	**Coef**	**S.E.**	**WALD Z**	**P value**	**Coef**	**S.E.**	**WALD Z**	**P value**	**Coef**	**S.E.**	**WALD Z**	**P value**	**Coef**	**S.E.**	**WALD Z**	**P value**
Immunosupression other	6.49	36.70	0.18	0.86	-0.60	0.59	-1.01	0.31	0.39	1.96	0.20	0.84	1.22	1.90	0.64	0.52
GCSF	-0.92	1.85	-0.50	0.62	-1.98	1.17	-1.69	0.09	-6.97	26.58	-0.26	0.79	-6.90	27.19	-0.25	0.80
Steroids	1.09	1.24	0.88	0.38	-0.01	0.62	-0.02	0.98	0.13	0.89	0.14	0.89	-0.49	0.86	-0.57	0.57
Previous Infection	1.34	0.26	5.18	<0.0001	1.60	0.21	7.66	<0.0001	1.73	0.25	6.87	<0.0001	1.54	0.24	6.30	<0.0001
Vaccine type (Pfizer)	1.60	0.30	5.32	<0.0001	0.95	0.22	4.40	<0.0001	1.51	0.26	5.69	<0.0001	1.67	0.26	6.34	<0.0001
Age	-0.02	0.01	-1.74	0.08	-0.02	0.01	-2.78	0.01	-0.03	0.01	-3.19	0.00	-0.01	0.01	-1.44	0.15

**ANOVA**	WT			Alpha			Beta			Delta		
**Factor**	**Chi-Square**	**d.f.**	**P value**	**Chi-Square**	**d.f.**	**P value**	**Chi-Square**	**d.f.**	**P**	**Chi-Square**	**d.f.**	**P value**
Immunosupression other	0.03	1	0.8597	1.02	1	0.3128	0.04	1	0.842	0.41	1	0.5201
GCSF	0.25	1	0.6187	2.84	1	0.0918	0.07	1	0.7932	0.06	1	0.7996
Steroids	0.77	1	0.3807	0	1	0.981	0.02	1	0.887	0.32	1	0.5706
Previous Infection	**26.85**	**1**	**<.0001**	**58.61**	**1**	**<.0001**	**47.25**	**1**	**<.0001**	**39.66**	**1**	**<.0001**
Vaccine type (Pfizer)	**28.3**	**1**	**<.0001**	**19.38**	**1**	**<.0001**	**32.38**	**1**	**<.0001**	**40.2**	**1**	**<.0001**
Age	3.04	1	0.0814	**7.73**	**1**	**0.0054**	**10.19**	**1**	**0.0014**	2.07	1	0.1507

NAbT were binned into three categories: Undetectable/low (IC50 titres <40), medium (40-256), high (>256)

NAbT, Neutralising antibody titers

CR, complete response

NACRTx, neoadjuvant therapy

NED, No evidence of disease

PD, Progressive disease

PR, Partial response

SD, Stable disease

GCSF, granulocyte colony-stimulating factor

Supplementary Table 8: Cohort characteristics Health care professionals

**Health care professionals, n=25**	
Age, median (IQR), years	44 (37-51)
Male, n (%)	3 (12)
**Covid Vaccine**	
AstraZeneca, n (%)	1 (4)
Pfizer, n (%)	22 (88)
Unknown, n (%)	2 (8)
Time to 2nd vaccine, median (IQR), days	78 (77-79)
Previous SARS-CoV-2 infection, n (%)	2 (8)

Supplementary Table 9: Concordance of T-cell and Nab response post-second dose

	WT Absent	WT Present	P	ALPHA Absent	ALPHA Present	P	BETA Absent	BETA Present	P	DELTA Absent	DELTA Present	P
						Patients with haematological malignancies						
**ELISPOT Absent**	8.3 (1)	21.4 (6)	ns	20 (4)	15.0 (3)	ns	25.00 (6)	6.3 (1)	ns	14.3 (3)	21.1 (4)	ns
**ELISPOT Present**	91.7 (11)	78.6 (22)		80 (16)	85.0 (17)		75.00 (18)	93.7 (15)		85.7 (18)	78.9 (15)	
						Patients with solid malignancies						
**ELISPOT Absent**	27.3 (3)	19.0 (33)	ns	25.6 (11)	17.6 (25)	ns	24.0 (12)	17.8 (24)	ns	23.6 (13)	17.7 (23)	ns
**ELISPOT Present**	72.7 (8)	81.0 (141)		74.4 (32)	82.4 (117)		76.0 (38)	82.2 (111)		76.4 (42)	82.3 (107)	
						Cancer patients						
**ELISPOT Absent**	17.4 (4)	19.3 (39)	ns	23.8 (15)	17.3 (28)	ns	24.3 (18)	16.6 (25)	ns	21.1 (16)	18,.1 (27)	ns
**ELISPOT Present**	82.6 (19)	80.7 (163)		76.2 (48)	82.7 (134)		75.7 (56)	83.4 (126)		78.9 (60)	81.9 (122)	

All values are shown as row-wise percentages, NAb with IC50 titres <40 was considered absent, T-cells were considered absent if SFU per million were <24. Significance was tested by Chi-Square test. WT, wild type
